# phylogatR: Phylogeographic data aggregation and repurposing

**DOI:** 10.1111/1755-0998.13673

**Published:** 2022-07-12

**Authors:** Tara A. Pelletier, Danielle J. Parsons, Sydney K. Decker, Stephanie Crouch, Eric Franz, Jeffery Ohrstrom, Bryan C. Carstens

**Affiliations:** ^1^ Department of Biology Radford University Radford Virginia USA; ^2^ Department of Evolution, Ecology, and Organismal Biology The Ohio State University Columbus Ohio USA; ^3^ Museum of Biological Diversity The Ohio State University Columbus Ohio USA; ^4^ Ohio Supercomputer Center Columbus Ohio USA

**Keywords:** biodiversity informatics, data repurposing, genetic diversity, macrogenetics, open science

## Abstract

Patterns of genetic diversity within species contain information the history of that species, including how they have responded to historical climate change and how easily the organism is able to disperse across its habitat. More than 40,000 phylogeographic and population genetic investigations have been published to date, each collecting genetic data from hundreds of samples. Despite these millions of data points, meta‐analyses are challenging because the synthesis of results across hundreds of studies, each using different methods and forms of analysis, is a daunting and time‐consuming task. It is more efficient to proceed by repurposing existing data and using automated data analysis. To facilitate data repurposing, we created a database (phylogatR) that aggregates data from different sources and conducts automated multiple sequence alignments and data curation to provide users with nearly ready‐to‐analyse sets of data for thousands of species. Two types of scientific research will be made easier by phylogatR: large meta‐analyses of thousands of species that can address classic questions in evolutionary biology and ecology, and student‐ or citizen‐ science based investigations that will introduce a broad range of people to the analysis of genetic data. phylogatR enhances the value of existing data via the creation of software and web‐based tools that enable these data to be recycled and reanalysed and increase accessibility to big data for research laboratories and classroom instructors with limited computational expertise and resources.

## 1 INTRODUCTION

Quantifying the geographic distribution of genetic variation within and between species provides essential information for understanding the evolutionary processes that give rise to current biodiversity patterns and is an essential aim of landscape genetic and phylogeographic investigations. The NCBI GenBank database houses over two hundred million DNA sequences, a number that grows monthly (https://www.ncbi.nlm.nih.gov/genbank/statistics/), but most of these sequences lack metadata associated with the locality from which the organism was collected. This limits the potential use of these data by preventing repurposing of the data (Sidlauskas et al., 2010) in any analysis that requires geospatial information. For example, Marques et al. (2013) found that only 7% of GenBank accessions of barcoding genes, such as *cytochrome oxidase I (COI)*, include latitude and longitude, and only 18% list museum catalogue information that can be used to link the sequence to a particular specimen. Similarly, Gratton et al. (2017) found that only 6.2% of GenBank tetrapod accessions include locality data. Overall, it has been suggested that 90% of biodiversity data remain unavailable for further use, and that missing geographic information was the most significant factor limiting use (Peterson et al., 2018). These “missing” locality data are particularly problematic when it is understood that voucher specimens from thousands of investigations are deposited into natural history collections, and that metadata associated with these vouchers, including in many cases georeferenced locality data, are currently available in other databases such as the Global Biodiversity Information Facility (GBIF).

Spatial information is extremely important to the biological sciences. For example, more than 22,000 published papers use some variant of the word “phylogeo*” in their title or abstract, in addition to more than 22,000 that use “population genetics” (https://www.webofscience.com/wos/woscc/basic‐search, 9 September 2021). These disciplines necessarily include spatial information, and this component enables researchers to explore topics such as speciation (e.g., Smith & Carstens, 2020), hybridization (Burbrink et al., 2021), demographic change (Carstens et al., 2018), and estimating the current (Farallo et al., 2020), former (Pelletier & Carstens, 2016) or future (Nottingham & Pelletier, 2021) species ranges, in addition to the evaluation of ecological niche overlap (Cavalcante et al., 2020). Given that researchers in each of these disciplines routinely collect sequence data from hundreds of samples (Garrick et al., 2015), the existence of georeferenced data in databases such as GenBank and Barcode of Life Database (BOLD) can enable novel comparative analyses.

Large‐scale meta‐analyses offer a promising strategy to understand the broad‐scale effects of geography, geology, and climate change on species distributions (Guralnick & Hill, 2009) and hold immense potential for insight (Dawson, 2014; Heberling et al., 2021). However, the considerable variation in study design and statistical analyses used across studies render meta‐analysis in population genetics and phylogeography difficult (Garrick et al., 2015). A more productive strategy is the repurposing of data (Blanchet et al., 2017; Leigh et al., 2021; Sidlauskas et al., 2010), where data from previously published work are reanalysed in large groups to extract insight about global processes. Combining similar types of data from multiple studies and then reanalysing these data under a common framework has the power to elucidate factors that drive evolution on both small and large scales.

One example of the potential of data repurposing is found in Miraldo et al. (2016). These researchers manually assembled mitochondrial DNA (mtDNA) sequences from almost 2000 species of terrestrial mammals and amphibians and used these data to document that genetic diversity is higher in the tropics and lower where human populations are high. This analysis required a considerable amount of effort, as data were mined by downloading GenBank and BOLD accessions that contained geographic coordinates or by emailing researchers to ask for their data. The data curation in Miraldo et al. (2016) was manual, which places an upper limit on the number of species that can be included in the analysis. More recent investigations have used automated computational pipelines to increase the efficiency of exploring population genetics and species limits on large scales in several ways. For example, Pelletier and Carstens (2018) used a Python script to assemble a database of over 8000 species of plants, fungi, and animals, analysed these data using R, and demonstrated that genetic structure within species was higher in northern latitudes and that the size of a species range was an important predictor of genetic structure.

Existing macrogenetic studies demonstrate the need for global analyses of genetic data. Large‐scale biodiversity data enhances conservation efforts (Pelletier et al., 2018; Thompson et al., 2021) and mapping the tree of life (Folk & Siniscalchi, 2021). There is a strong push for making data publicly available (Marden et al., 2021) and repurposing these data increases their value (Heberling et al., 2021; Whitlock et al., 2010). It opens the doors for reexamining classic questions on larger scales, but also moves forward the fields of population genetics, phylogeography, and systematics by increasing the power to tease apart the complex processes that shape biodiversity patterns (Hickerson et al., 2010; Papadopoulou & Knowles, 2016). Furthermore, these field are increasingly integrating data types (e.g., environmental data layers, morphological measurements, life history characteristics) with large‐scale genetic and geographic data, which will not only enhance our understanding of the ecological processes that contribute to evolutionary change, but also provide applicable information for conservation purposes (Anderson et al., 2020).

In order to facilitate phylogeographic analyses on the largest possible scale (i.e., continental or global) from thousands of species, we have developed software that parses accessions from several repositories of geographic and genetic information, organizes them into a common framework under a taxonomic hierarchy, and produces multiple sequence alignments that are ready to be analysed. Our goal was to develop a database that is user‐friendly and accessible to researchers and instructors without much training in computational biology whose efforts are aimed at conducting studies on specific taxonomic groups and/or biogeographic regions. This effort contributes to Findability, Accessibility, Interoperability, and Reusability (FAIR) initiatives that aim to improve the infrastructure of open‐data science (Heberling et al., 2021; Wilkinson et al., 2016). The database, phylogatR (phylogeographic data aggregation and repurposing) is freely available via the Ohio Supercomputer Center (OSC), along with several R scripts to aid in data curation, analysis, and education.

## 2 phylogatR PIPELINE

**FIGURE 1:**
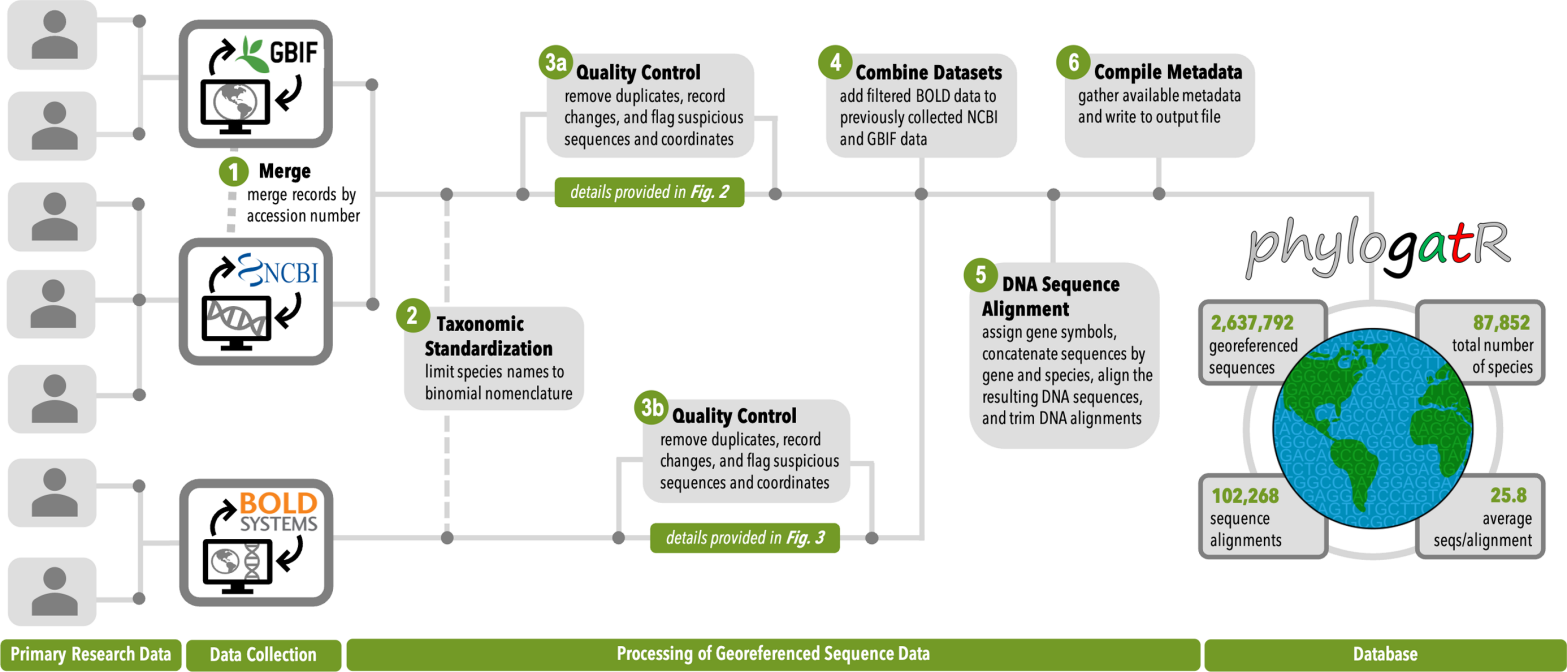
Overview of phylogatR pipeline

Data for our aggregator comes primarily from three large databases. (1) GBIF (https://www.gbif.org/), an open‐source database funded and supported since 1999 by a large group of government agencies worldwide. It contains over two billion occurrence records from over 6 million organisms across the globe. (2) NCBI GenBank, a collection of DNA sequence data from three organizations: DNA DataBank of Japan (DDBJ), the European Nucleotide Archive (ENA), and GenBank at NCBI, (https://www.ncbi.nlm.nih.gov/genbank/), and (3) BOLD http://www.boldsystems.org/index.php), developed by the Center for Biodiversity Genomics in Canada, contains barcode data for almost 600,000 species. Pipeline choices were made to minimize data duplication and loss, conduct preliminary cleaning and alignment, and to return results to users in a manner that is transparent and enables them to conduct additional curation as needed. Scripts for data aggregation and cleaning are available in our GitHub repository (https://github.com/OSC/phylogatr‐web). A schematic overview of the pipeline is available in Figure 1.

### 2.1 Data aggregation

**FIGURE 2:**
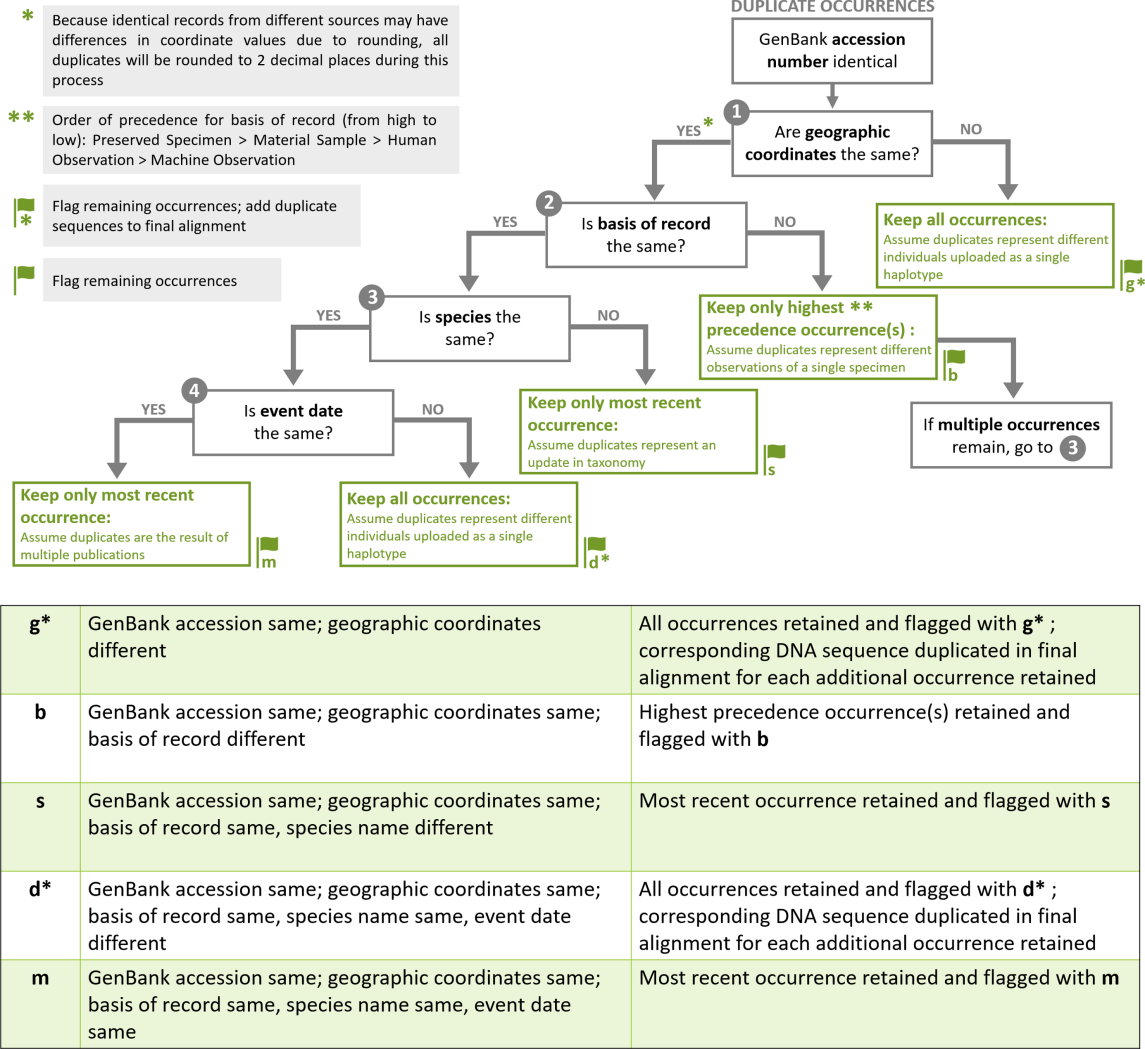
Data curation steps for GBIF and GenBank data

**FIGURE 3:**
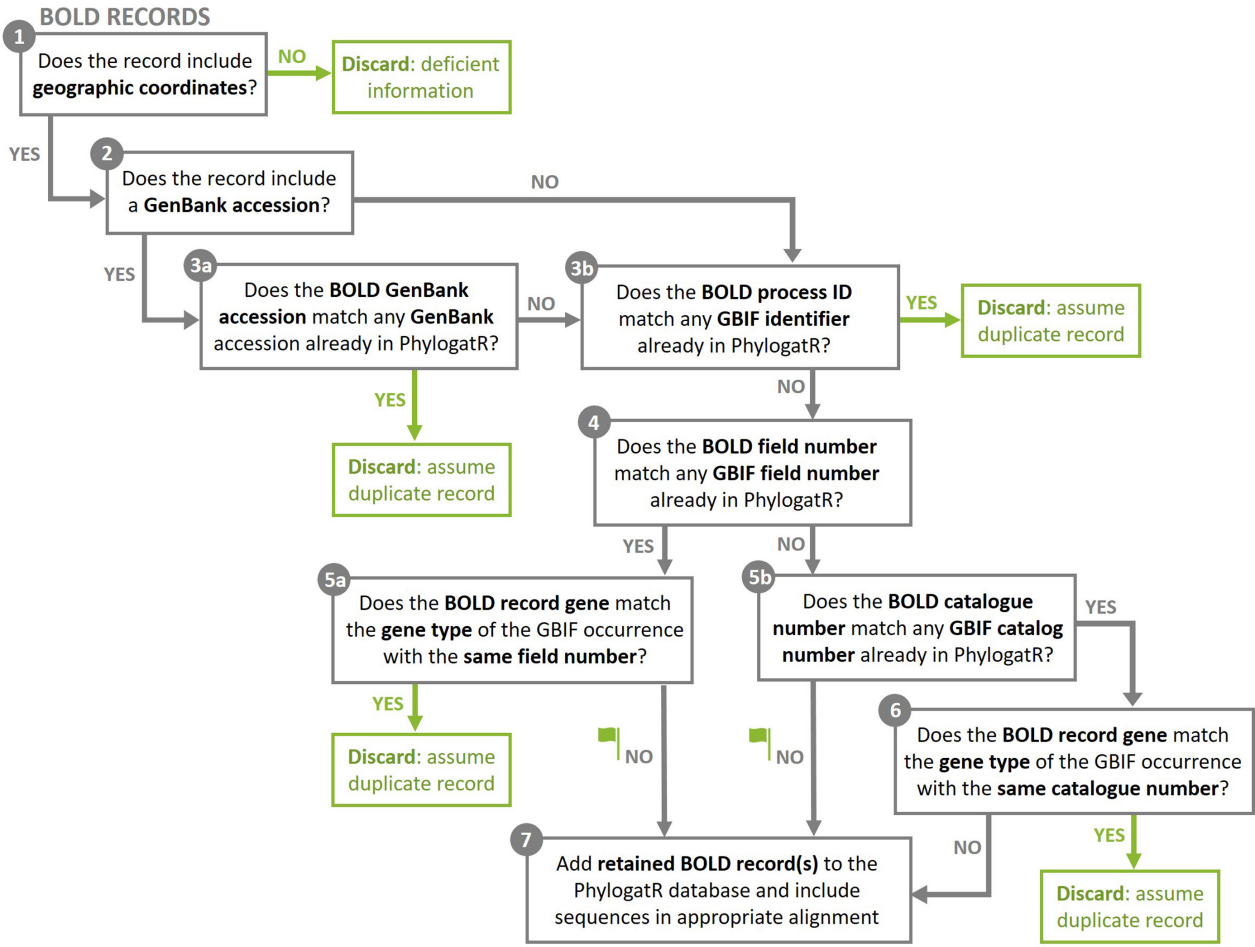
Data curation steps for BOLD data

Data were downloaded from GBIF that included coordinates, excluding those flagged as suspicious, contained sequence accessions, and a full binomial name. We only included occurrences in which Basis of record was either PreservedSpecimen, MaterialSample, HumanObservation, or MachineObservation. The entire GenBank nucleotide sequence database was downloaded using the rsync file transfer program. Occurrences and DNA sequences that contained the same GenBank accession were matched and curated (Figure 2). For each occurrence, sequence accessions and geographic coordinates were checked for duplication. First, all coordinates were rounded to two decimals to overcome differences in coordinates that come from the same sample but appear different due to rounding. If coordinates were different, but had the same GenBank accession, we assumed duplicates represent different individuals uploaded to GenBank as a single haplotype. In this case, all occurrences were kept, but each was flagged with “**g**” so that users can explore these accessions if necessary. If coordinates were the same, we checked the basis of record. If these were different, we kept only the highest precedence for an observation (from high to low: preserved specimen, material sample, human observation, machine observation), with the assumption that these sequences with the same GenBank accession and geographic coordinates was a different observation of the same specimen, and each was flagged with “**b**”. If basis of record was the same, we checked the species name. If different, we assumed a change in taxonomy and kept the most recent occurrence and flagged it with “**s**”. If the species name was also the same, we checked the event date. If different, we assume the duplicates represent different individuals, and they were flagged with “**d**”, again to allow further investigation by users. For any duplicates that had the same GenBank accession, geographic coordinates, species name, and event date, but different GBIF occurrences, we retained only the most recent occurrence and flagged with “**m**”. Next, the BOLD database was scraped to obtain taxon names and data were pulled by looping through 500 taxa at a time using the public API. All available data were downloaded and curated (Figure 3). Records without coordinates were removed. Those with GenBank or GBIF accessions already in our database were removed.

We standardized gene and species names to the best of our ability. For example, we assigned a common gene symbol for commonly sequenced genes that are often represented by more than one symbol (Table S1), such as *COI* for *cytochrome oxidase I* that is also often depicted as *COXI* or *CO1*. In some cases, genes were identified with a different gene symbol but the same gene name. These were left alone assuming that they represent different regions of the same gene, such as the *malic‐enzyme* that contains alignments for *ME1* and *ME2*. While we expect few instances where these gene symbols are incorrect, we advise users to scan the list of genes in their dataset before use. Species names were limited to binomial nomenclature, though those with subspecies identifiers are listed in the associated metadata. GBIF taxonomy was retained when it did not match the GenBank taxonomy and these are also flagged in the associated metadata. We recommend individual users to capitalize on available tools for checking taxonomy when appropriate for their needs. For example, the R package taxize (Chamberlain & Szöcs, 2013) accesses many data sources to update species names, or standardized databases can be used directly to update species names such as the Mammal Diversity Database published by the American Society of Mammalogists (as in Parsons et al., 2022).

### 2.2 Multiple sequence alignment

Every sequence is identified by species, gene, GenBank accession, GBIF ID, and/or BOLD ID. All sequences were concatenated based on identical gene sequence symbol and species name. We conducted multiple sequence alignments for all genes where there were at least three sequences within a species on a species‐by‐species basis. First, the default MAFFT version 7 parameters were used. Sequence alignments were checked by eye for 10 families (117 species‐level alignments) that were previously determined to require post‐alignment adjustments (Parsons et al., 2022). Several alignments were found to have large sequence gaps at the ends of the alignment, while others contained unwanted sequences (e.g., parasitic sequences that have been named as the host species). After this first round of checking, only eight alignments needed trimming and three needed sequences removed (or reverse complimented). We updated the MAFFT settings to include the adjustdirection and inputorder features. Then trimAl version 1.2 was used to clean the alignments. After several iterations of parameter settings, we set resoverlap to 0.85, seqoverlap to 50, and gt to 0.15. Identical sequences (same GenBank accession) with multiple GBIF occurrences that have been deemed not duplicates (Figure 2) are repeated for the final sequence alignment. While these settings appear to eliminate most issues that arise from within species sequence alignments, researchers should screen their data for outliers before data analysis. We suspect these issues to be minimal, and when dealing with large datasets a small amount of noise is not expected to alter results (see Section 3 below).

### 2.3 Data

The database currently contains 87,852 species and 102,268 sequence alignments. The average number of alignments per species is 1.2 and the average number of sequences per alignment is 25.8. The database includes species from Animalia (77,743), Plantae (7905), Fungi (1971), Chromista (229), and Protozoa (4). Out of the almost two billion GBIF occurrences, 1.6 billion contained geographic coordinates and matched our search filters. We retained about 10.5 million with genetic accessions to run through our pipeline, the majority of which were removed during data cleaning steps. After downloading just over 1.3 million records from BOLD, about 500,000 sequences were retained which included geographic coordinates, valid IDs, and were not duplicates. The final database contains over 2.6 million records. Most of the data are from mitochondrial and chloroplast DNA, a result that reflects the key role of genes from these organellar genomes to disciplines such as phylogeography (Garrick et al., 2015). After merging genes with different known gene symbols, our database contains a total of 1988 genes. Note that phylogatR has been designed to be expandable and will grow by rerunning the pipeline each month to add new accessions from GenBank, GBIF, BOLD, and potentially other sources for at least 10 years, and updates and fixes will be made as identified.

When data are downloaded from phylogatR (zip and tarball formats are available), all data are nested within directories that are structured by taxonomic rank. Each species folder consists of an unaligned fasta file (extension *.fa)* and an aligned fasta file (extension *.afa*) for each locus available for that species. Each species folder also contains an occurrence file that contains the original database accessions and geographic coordinates in decimal form, as well as any appropriate flags. The root folder contains information for each sequence alignment (in the *genes.txt* file), including the number of sequences before and after data cleaning steps, taxonomic information, and flags those that may contain inconsistencies in species names across databases. The database is available at https://phylogatr.org/. An indicated shortcoming of current biodiversity data aggregators is the lack of back and forth communication between primary producers of data, data aggregators, and end‐users (Anderson et al., 2020). We provide a means for submitting feedback and suggesting edits and data flags via an email address (Phylogatr@lists.osu.edu) that is reviewed by the team of biologists and computer programmers. We also include R tutorials for checking data before formal analyses begin.

## 3 EMPIRICAL EXAMPLE

We explored how genetic diversity is correlated with range size in almost 80,000 species and over 2 million sequences from the database (Table 1). Many measures of genetic diversity exist and can be used to understand different aspects about an individual, population, species, or community. By looking at patterns in genetic diversity, inferences can be made regarding evolutionary processes like migration, selection, and drift, and is often a first step in most genetic studies. Several measures of genetic diversity exist that capture different aspects of the data, such as estimates of the number of segregating sites (S), the number of haplotypes (H), and the mean per‐site pairwise number of nucleotide differences between sequences (π). It is expected that widespread species would have higher genetic diversity due to their (presumed) larger population sizes (Young et al., 2006). Custom R version 4.0.4 (R Core Team, 2020) scripts were used to analyse data from several taxonomic groups by downloading sequence alignments by taxonomic group from the phylogatR database between 18 May 2021 and 11 June 2021.

**TABLE 1:** Summary of data downloaded from the database for analysis

Kingdom	Phylum	Class	Order	Common name	*n* Species downloaded	*n* Alignments downloaded	*n* Species	*n* Alignments	Mean genes per species	Mean sequences per alignment
Animalia	Chordata	Actinopterygii		Ray‐finned fishes	7629	8445	7629	8445	1.1	15.1
		Amphibia		Frogs, salamanders, ceacilians	719	1296	719	1296	1.8	16.02
		Aves		Birds	2409	2828	2409	2828	1.1	15.3
		Elasmobranchii		Sharks, rays, skates	436	463	436	463	1.1	13.1
		Mammalia		Mammals	1000	1672	1000	1672	1.7	26.8
		Reptilia		Turtles, crocs, snakes, lizards	767	1171	767	1171	1.5	13.1
	Annelida			Earthworms, leeches	664	747	664	747	1.1	16.6
	Arthropoda	Arachnida		Spiders, scorpions, ticks, mites	2326	2484	2326	2484	1.1	34.8
		Insecta								
			Hymenoptera	Ants, bees, wasps	7656	7865	7656	7865	1.02	23.9
			Coleoptera	Beetles	5669	5804	5669	5804	1.02	17.8
			Lepidoptera	Butterflies, moths	27,005	27,292	27,005	27,292	1.01	20.5
			Diptera	Flies, mosquitoes	8316	9074	8316	9074	1.1	55.4
			Orthoptera	Grasshoppers, crickets, roaches	519	598	519	598	1.2	15.3
			Odonata	Dragonflies	461	489	461	489	1.1	13.3
		Malacostraca		Crabs, lobsters, shrimp	1733	1940	1733	1940	1.1	20.1
	Cnidaria			Jellyfish, sea anemones, coral	281	404	281	398	1.4	11
	Mollusca	Bivalvia		Clams, oysters, mussels, scallops	365	459	365	459	1.3	31.9
		Cephalopoda		Octopus, squids, cuttlefish	102	119	102	119	1.2	15.5
		Gastropoda		Snails, slugs, conchs	1330	1657	1330	1657	1.2	17.9
	Nematoda			Roundworms	135	145	135	134	1.1	23.2
	Platyhelminthes			Flatworms	143	186	143	186	1.3	19.8
	Porifera			Sea sponges	45	50	45	50	1.1	8.8
Fungi	Ascomycota			Sac fungi (yeast)	840	1187	838	1186	1.3	10.2
	Basidiomycota			Club fungi (mushrooms)	1107	1240	1107	1240	1.1	6.3
Plantae	Bryophyta			Mosses, liverwarts	40	96	40	96	2.4	7.4
	Chlorophyta			Green algae	88	148	88	148	1.5	15.7
	Lycopodiophyta			Clubmoss, spikemoss	10	14	10	14	1	3.6
	Pinophyta			Pines, conifers	14	16	14	16	1.1	4.3
	Rhodophyta			Red algae	609	938	609	938	1.5	16.2
	Tracheophyta			Sea grasses	6189	13,250	6189	13,250	2.1	7.3
	Magnoliophyta				926	1399	926	1399	1.5	4.3
				Totals	79,533	93,476	79,531	93,458	1.3	16.8

*Note:* Downloads were conducted by the lowest taxonomic group listed in the table. The number of species and alignments are those that were included in the data analysis pipeline before and after checking for binomial nomenclature and genetic or geographic outliers.

First, species names were scanned using the *genes.txt* files to find typos in species names, as well as other abnormalities in naming patterns. In several groups there were some nonbinomial naming patterns (Table S2). In the Platyhelminthes, Nematodes, Bivalves, Elasmobranchs, Hymenoptera, Lepidoptera, Diptera, Malacostraca, and Chlorophyta, there were several Genera that included species names labelled as letters and/or roman numerals (e.g., Co*tylurus c* or *f*.; *Paratylenchus BITH I* or *II*; *Hiatella C* or *D; Squalus clade B* or *C*; *Braunsapis A* or *B; Adoxophyes C* or *D*; *Allograpta CR A* or *B*; *Uristes murrayi morphospecies A* or *B; Ostreonium TeA* or *TeF*). In these instances, taxonomic expertise will be needed in deciding whether to treat these as different species. In one case there seemed to be an indication of a lateral gene transfer in Tracheophyta, which would need to be treated with caution (*Alloteropsis semialata* PCK 1P1 LGT:C and PPC 1P3 LGT:M). In another case, there was a misspelling in a name that we have updated in the database. This is an area of work where we are seeking user input but overall, the level of errors detected based on our exploration of these data are quite low, and easily checked by eye. The regression analysis below was carried out on the data with and without these abnormalities removed, and none had a significant impact on the results.

Nucleotide diversity (π) was calculated for each sequence alignment using the nuc.div function from the R package pegas (Paradis, 2010). Geographic coordinates from each species were used to estimate the range of the species, though this only represents the sampling range of a species. It is important to note that when interpreting these data, they may not encompass the full range of a species, as indicated by the large number of GBIF occurrences that do not include sequence data. Scatter plots of area and π were created for each taxonomic group using the package ggplot2 (Wickham, 2017) to examine the data visually for outliers (Table S3 and Supporting Information Figures). When outliers were detected by area, online distribution maps were compared to the geographic coordinates from the data set. In all these cases (58 total), the coordinates fell within the known published distributions. In cases where outliers were detected by π (23 total), the geographic coordinates were also checked according to the published distributions. Again, no points fell outside the published distributions. These sequence alignments were also checked for possible mis‐identified sequences or poorly aligned sequences. In most cases, a sequence or two slipped through our data cleaning steps and probably does not belong to either that species or locus and therefor produced a poor sequence alignment. The regression analysis below was conducted with and without the π and area outliers removed, and none had a significant impact on the results (Table S3; https://phylogatr.org/assets/modules/phylogatR_genetic‐diversity.html).

Several other sequence alignments from our initial download were not included in the following analyses (Table S4). These alignments produced NA values for π (1050 total) and were explored further. In the majority (~95%) of cases, different portions of a given gene were sequenced such that there was no overlap in the middle of the sequence. In these instances, it is incumbent on the user to determine whether this level of missing data is appropriate for their analysis. The remaining cases were attributed to poor sequence alignments, usually due to just one sequence passing through our data cleaning steps. As such cases are discovered, alignments will be manually curated and updated in the database. As bad alignments are discovered, user input via the help documentation is encouraged. As updates become necessary, we will capture all manual corrections in a log file akin to a write‐ahead‐log. This log will hold all the records before and after any manual edit, including the date of change, and sql commands executed to make the change. This information will then be parsed and added to the website, including user flags that have not been incorporated into the database.

Regression analysis was conducted to determine whether the size of geographic sampling could explain variation in genetic diversity using the lm function in R. Since we conducted 31 regression analyses, a Bonferroni correction was used to adjust our *p*‐value (.05/31 = 0.0016). Ten out of the 31 tests were significant (Table 2). In the vertebrates, only Actinopterygii, Elasmobranchii, and Mammalia were significant. In the insects, the Hymenoptera, Coleoptera, Lepidoptera, and Orthoptera, were significant. Porifera was significant, along with two plant groups (Rhodophyta and Tracheophyta). Only Porifera stands out as having a particularly high R‐square value, while the others, while significant, were quite low. These numbers for Porifera may be driven by one species with particularly high π and area, however, when this species is removed, the relationship is still significant (*p* < .0001) and *R*‐square drops from .78 to .43 (Table S3; Supporting Information Figures). Otherwise, no patterns emerge as far as which taxonomic groups would be more likely to display a relationship between area and π, or whether being winged, terrestrial, etc., for example, would contribute to an increase or decrease in genetic diversity, given the size of a species geographic distribution. There are probably a combination of factors that contribute to levels of genetic diversity within a species. It might be useful to explore how sampling effort influences the measures of genetic diversity we can estimate based on available data (i.e., does genetic sampling accurately reflect the distribution of a species?). This analysis is only a first step towards understanding how life history and dispersal ability may contribute to genetic variation and population structure globally.

**TABLE 2:** Summary of linear regression results

Group	Pi (mean)	*R*‐square	*p*‐Value
Actinopterygii	0.0165	.0085	**2.20E‐16**
Amphibia	0.0258	−.0007	.776
Aves	0.0056	−.0014	.8495
Elasmobranchii	0.0100	.0274	**.0002**
Mammalia	0.0218	.0134	**1.44E‐06**
Reptilia	0.0491	−.0002	.3644
Annelida	0.0361	.0041	.0448
Arachnida	0.0150	.0021	.0034
Hymenoptera	0.0187	.0020	**5.09E‐05**
Coleoptera	0.0132	.0038	**5.74E‐07**
Lepidoptera	0.0104	.0033	**2.20E‐16**
Diptera	0.0140	.0000	.4218
Orthoptera	0.0147	.0153	**.0014**
Odonata	0.0398	−.0019	.7434
Malacostraca	0.0279	.0008	.1069
Cnidaria	0.0376	.0035	.2394
Bivalvia	0.0245	.0173	.0027
Cephalopoda	0.0138	.0208	.0637
Gastropoda	0.0260	−.0006	.8771
Nematoda	0.0219	.0032	.2276
Platyhelminthes	0.0270	−.0055	.9588
Porifera	0.0141	.7833	**2.20E‐16**
Ascomycota	0.0132	−.0010	.6397
Basidiomycota	0.0183	.0054	.0065
Bryophyta	0.0104	−.0092	.7178
Chlorophyta	0.0349	−.0012	.3605
Lycopodiophyta	0.1067	−.0504	.4725
Pinophyta	0.1989	.1868	.0534
Rhodophyta	0.0081	.0258	**5.24E‐07**
Tracheophyta	0.0236	.0109	**2.20E‐16**
Magnoliophyta	0.0394	−.0004	.5011

*Note:* A Bonferroni correction was used to adjust our *p*‐value (0.05/31 = 0.0016). Those that are significant are in bold.

Two plant groups have relatively high values for π (e.g., Lycopodiophyta, Pinophyta). This suggests these groups are worth further exploration, as either they may be in need of database updates to reflect taxonomic revisions and misidentifications, or these groups may harbour a high number of cryptic species (Parsons et al., 2022). Additionally, though still highlighting the need for further work, this could be a sampling issue as these groups had lower species representation in the database and we might be misrepresenting the average. Future studies could explore how sampling effort of species numbers influences average measures of genetic diversity such as ours. Documenting global levels of genetic diversity, an important measure of biodiversity, can serve as a baseline for detecting rapid changes, or loss of diversity, due to climate change (Paz‐Vinas et al., 2018). Furthermore, measures of genetic variation are often used to assess the ability of a species or population to respond to environmental (climate, habitat, biotic) changes (Frankham, 2005; Hoffmann & Sgrò, 2011); large‐scale analyses such as this, allow for targeting individual species that might be at a higher risk for extinction (Frankham et al., 2014; Hoban et al., 2020) and for identifying species attributes that contribute to higher levels of genetic diversity (Broadhurst et al., 2017). While there is no consensus as to whether measures of genetic diversity from a single mitochondrial or chloroplast gene, the most common in our dataset, are appropriate measures of genetic diversity (Paz‐Vinas et al., 2021; Petit‐Marty et al., 2021), many species (15%; 13,960 total) have data from multiple loci in phylogatR and measures of genetic diversity across loci can be evaluated. However, including spatial information for individuals allows further insight into the factors that contribute to increasing or decreasing levels of genetic variation, such as shared barriers to dispersal and responses to environmental change. Genetic diversity alone may not be a strong indicator of species stability but integrating the information that can be gained via geographic coordinates (e.g., climate layers) is necessary to consider demographic history and environmental variables for implementing effective conservation strategies (Teixeira & Huber, 2021).

A useful secondary product of the analysis described above is the opportunity to explore outliers and inconsistencies in the database. We identified alignments (1.2% of the data) that could potentially bias our results. While in our case there is sufficient data that a small amount of noise caused by outliers and inconsistent species names did not influence the results (Tables S2–S4), this may not be universally true for all analyses. We had 1,511,882 occurrences with flags (Table S5). Of those that were flagged, the majority of these were flag “**g**” (50%), where the GenBank accessions are the same, but geographic coordinates are different, followed by flag “**d**” (18%), where GenkBank accession, geographic coordinates, basis of record, and species name are the same, but the event date is different, suggesting many historical DNA sequences had been uploaded to GenBank as haplotypes. We recommend those uploading data to these databases refrain from uploading haplotypic data and include DNA sequences from all individuals or indicating on GBIF that data from GenBank represent haplotypic data. Flag “**b**” (26%), where GenBank accessions and geographic coordinates are identical but the basis of record is different, and flag “**m**” (4.8%), where GenBank accessions, geographic coordinates, basis of record, species name, and event date are all the same, were the next most common flags, suggesting there are many duplicates in these databases that need to be removed. Finally, flag “**s**” occurred in only 0.2% of flagged occurrences, where GenBank accessions, geographic coordinates, and basis of record are the same, but species names are different, indicating that taxonomy issues are present, but do not overwhelm the data. Users of phylogatR are cautioned to pay attention to flagged sequences and alignments and to make appropriate corrections as dictated by the needs of their investigation protocol. The scripts used for these analyses are available on the phylogatR website and can be used to facilitate screening the data.

The exploration of these data began in a bioinformatics course that aimed to introduce students to multiple sequence alignments, highlight the value of estimating genetic diversity and using open‐source databases, and learn the structure of creating loops. This work was completed due to efforts from one of these undergraduate researchers (S. Crouch), who led the analysis of these data for this empirical example. The datasets that can be generated via phylogatR will contribute to the ongoing development of resources that will expose students to real data and computational methods in the classroom. Incorporating authentic research into classroom instruction provides inclusive learning experiences for all students and leads to better learning outcomes (Theobald et al., 2020). The additional benefit of phylogatR is that concepts in evolution and ecology can be taught with real data at no cost, other than computer access. The phylogatR website contains teacher resources, which include teaching modules and associated instructor notes, with intent to increase these resources in the future.

## 4 CONCLUSIONS

Identifying the evolutionary and environmental processes that have influenced a single lineage is an ongoing practice for evolutionary biologists, but a true understanding of these processes will require the synthesis of results from thousands of individual studies. Such a synthesis will be most efficiently achieved via data repurposing and automated analysis. phylogatR makes such syntheses more accessible for all researchers. By bypassing the idiosyncratic results of individual studies, phylogatR will enable biologists to test hypotheses at various taxonomic and geographic scales. The example analysis presented above combines genetic and geographic data in a way that is only meaningful when done on a large scale. These results indicate that we will make fundamental contributions to understanding global patterns of genetic diversity that will have important implications to conservation management and species discovery (see Table 3).

**TABLE 3:** The phylogatR database and web portal can enable the testing of these hypotheses (among others) on a continental or global scale

Hypothesis	Example references
Current ecological communities are historically stable	Zink (2002)
Shared organismal traits lead to concordant phylogeographic patterns	Papadopoulou and Knowles (2015) and Zamudio et al. (2016)
Members of ecologically‐interdependent communities will codiversify	Smith et al. (2011) and Satler and Carstens (2016)
Pleistocene refugia are shared by species from many taxonomic groups	Brunsfeld et al. (2001)
Cosmopolitan species will have higher levels of genetic diversity than small endemics	Gitzendanner and Soltis (2000)
Regions of marginal habitat contain less genetic diversity	Micheletti and Storfer (2015)
Generalist species will have weaker responses to climatic and landscape changes than habitat specialists	Estavillo et al. (2013)
Southern peninsular regions served as Pleistocene refugia in the Northern Hemisphere	Hewitt (1996)
Cryptic species are likely to be present in regions of high endemism	Reeder et al. (2007)
Ecological niche differentiation promotes genetic diversification	McCormack et al. (2010)
Historical demographic processes are shared among species encountering the same changes in climate	Hewitt (2004)

While single‐locus data has its limitations in making inferences about historical demography (Matumba et al., 2020), DNA barcoding, or the use of other single‐locus DNA markers, has provided tremendous insight into identifying evolutionary significant units and providing information on species in further need of exploration (Bousjein et al., 2021; León‐Tapia, 2021; Nneji et al., 2020; Sholihah et al., 2020; Wang et al., 2020). These data are particularly helpful when aiming to explore broad‐scale patterns such as those on a continental scale (Dincă et al., 2021) or across species (Doorenweerd et al., 2020), especially for a large number of taxonomic groups, as demonstrated here. Studies using data from our initial data aggregation pipelines have further demonstrated the utility of single‐locus large‐scale studies that also utilize data layers from other sources. Parsons et al. (2022) explore cryptic diversity in mammals using molecular species delimitation methods for single‐locus data in conjunction with natural history and environmental data for over 4000 species. They found that hundreds of mammal species are still probably undescribed and that these are mostly small‐bodied taxa with large ranges (scripts for this project can be found at https://github.com/parsons463/HiddenDiversity).

Our brief empirical example allowed us to document outliers in the data and search for poor sequence alignments, as we will continue to improve the database and data curation steps. We will continue to make recommendations and supply users with guidance in data checking before analysis. We encourage continued natural history work to better populate biodiversity databases as the benefits of publicly available data are numerous and experts are needed to correct database errors and decide where data deficiencies lie (Groom et al., 2020; Leigh et al., 2021). Further, making data easy to access and reuse is important for researchers and educators who do not have the skills or resources for large‐scale projects, or expensive and time‐consuming field and laboratory work, increasing participation from underprivileged groups and minorities (Estrada et al., 2016; Hudson et al., 2020; Whittington & Pelletier, 2021). By making real genetic data available to students from any school with a connection to the internet, phylogatR will inspire the next generation of researchers to understand and protect biodiversity while they are developing the computational skills that are increasingly required for evolutionary and ecological studies. Not only do these data make authentic research more readily available in the classroom, they increase the access to biodiversity data worldwide, therefore contributing to a more inclusive and diverse STEM community and easily implemented international collaborations (Heberling et al., 2021; Marden et al., 2021).

Perfect data is unattainable and not all data will be retained after data curations steps (Peterson et al., 2018). The data currently available on phylogatR offer a first step towards asking big questions with big data in population genetics, phylogeography, and systematics. While this study does not aim to solve problems in data standards, making data more readily available will probably result in novel questions and transformative findings, and will largely contribute to identifying current shortcomings and inconsistencies in current data sharing practices. We expect that this effort will increase the desire for aggregating next‐generation data to obtain multi‐locus sequences from a large number of species in order to ask even more refined questions in phylogeography on a global scale.

## AUTHOR CONTRIBUTIONS

Tara A. Pelletier: designed research, performed research, wrote the manuscript, acquired funding. Bryan C. Carstens: designed research, performed research, wrote the manuscript, acquired funding. Danielle J. Parsons: performed research, edited the manuscript, created figures. Sydney K. Decker: performed research, edited the manuscript, created logo. Stephanie Crouch: analysed data, edited paper. Eric Franz: contributed code, edited the manuscript. Jeffery Ohrstrom: contributed code, edited the manuscript.

## CONFLICTS OF INTEREST

The authors declare no conflicts of interest.

### OPEN RESEARCH BADGES

This article has earned an Open Data badge for making publicly available the digitally‐shareable data necessary to reproduce the reported results. The data is available at 10.5061/dryad.bzkh1899x.

## BENEFIT‐SHARING STATEMENT

Benefits from this research include accessibility to big data via the public database, minimizing the need for computational resources, as described above, which include data analysis pipelines and educational tools.

## Supporting information


Appendix S1

Appendix S2

## Data Availability

The phylogatR database is publicly available at https://phylogatr.org/ where every download includes the GBIF DOI, GenBank version, and BOLD DOI that contributed to the data. All scripts devoted to the development of the database can be found at https://github.com/OSC/phylogatr‐web. Scripts and data files used for the empirical example can be found on DRYAD doi:10.5061/dryad.bzkh1899x.
