# Supplementary material for: phylogatR: Phylogeographic data aggregation and repurposing
Source: Mol Ecol Resour. 2022 Jul 12;22(8):2830–42. doi: 10.1111/1755-0998.13673 (PMC9796472; doi:10.1111/1755-0998.13673)
Supplement: Supplementary file 1 — Appendix S1 [file MEN-22-2830-s002.pdf]

Supplemental figures for

phylogatR: Phylogeographic data aggregation and repurposing

Tara A Pelletier[1*], Danielle J Parsons[23], Sydney K Decker[23], Stephanie Crouch[1], Eric Franz[4], Jeffery Ohrstrom[4], Bryan C Carstens[23]

[1]Department of Biology, Radford University, Radford, VA, 24142
[2]Department of Evolution, Ecology, and Organismal Biology, The Ohio State University, Columbus OH, 43210
[3]Museum of Biological Diversity, The Ohio State University, Columbus, OH, 43212
[4]Ohio Supercomputer Center, Columbus OH, 43212
[*]corresponding author: tpelletier@radford.edu

Regression plots for all taxonomic groups

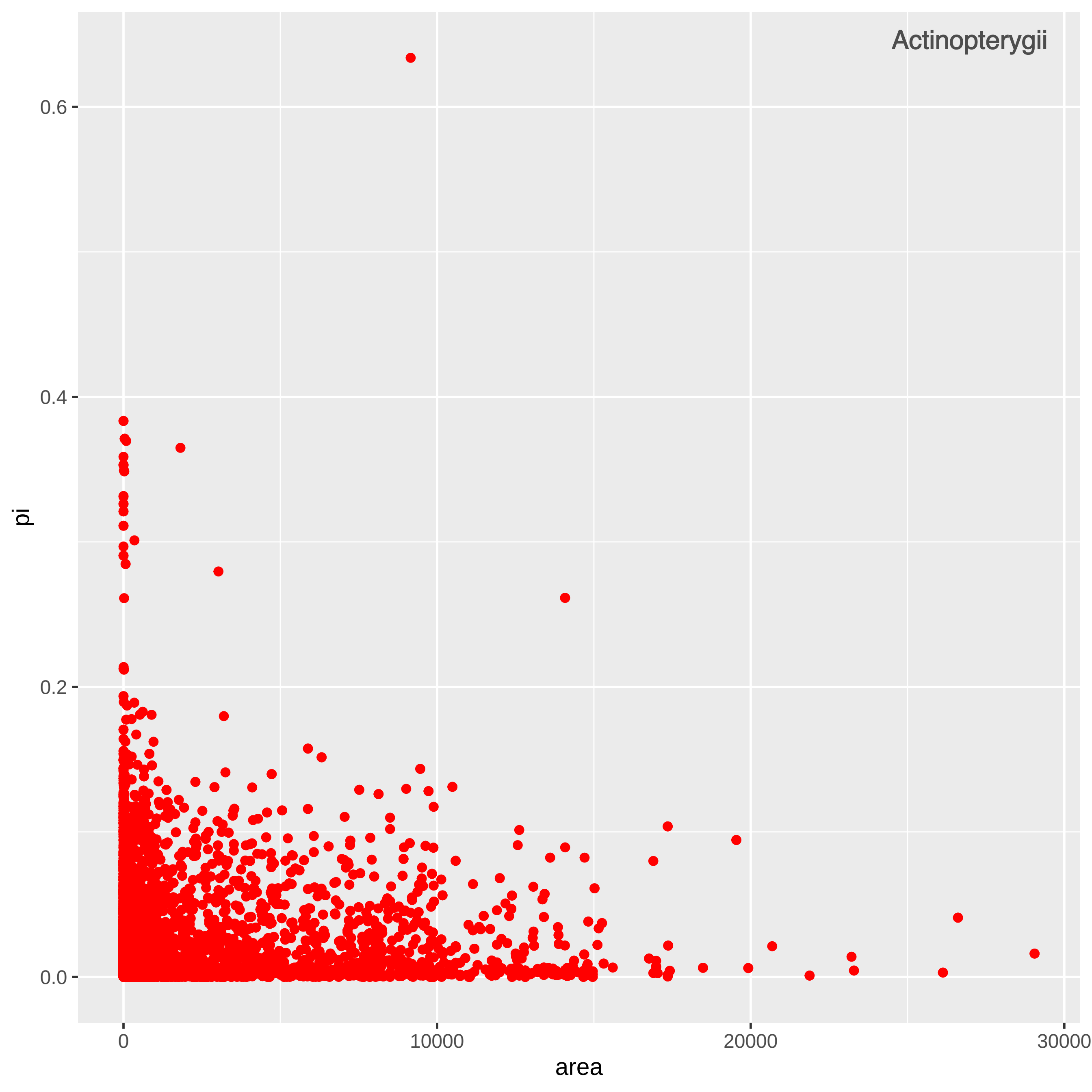
Actinopterygii
0.6
0.4
0.2
0.0
pi
0
10000
20000
30000
area

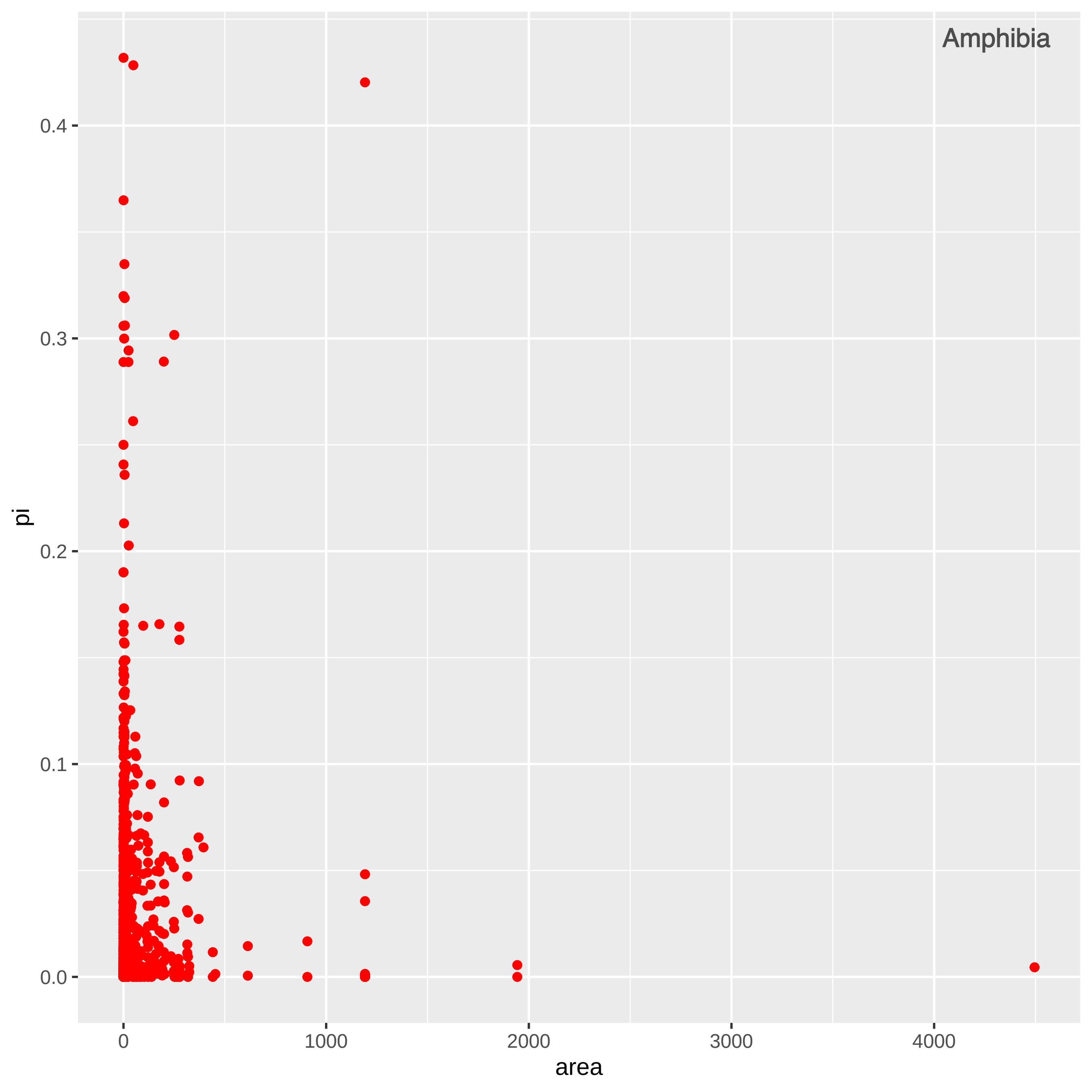

Amphibia
pi
0.0
0.1
0.2
0.3
0.4
area
0
1000
2000
3000
4000

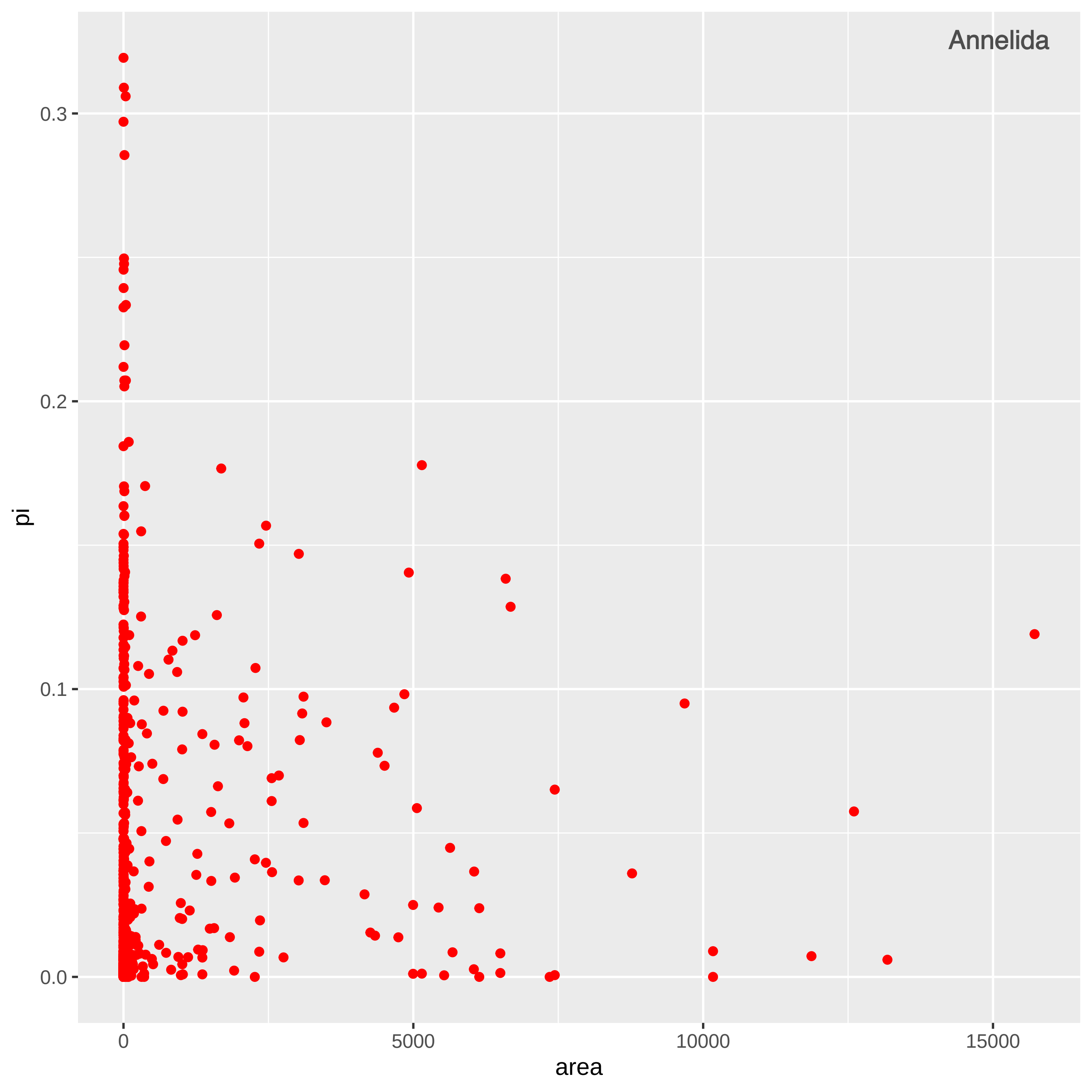

Annelida
0.3
0.2
0.1
0.0
pi
0
5000
10000
15000
area

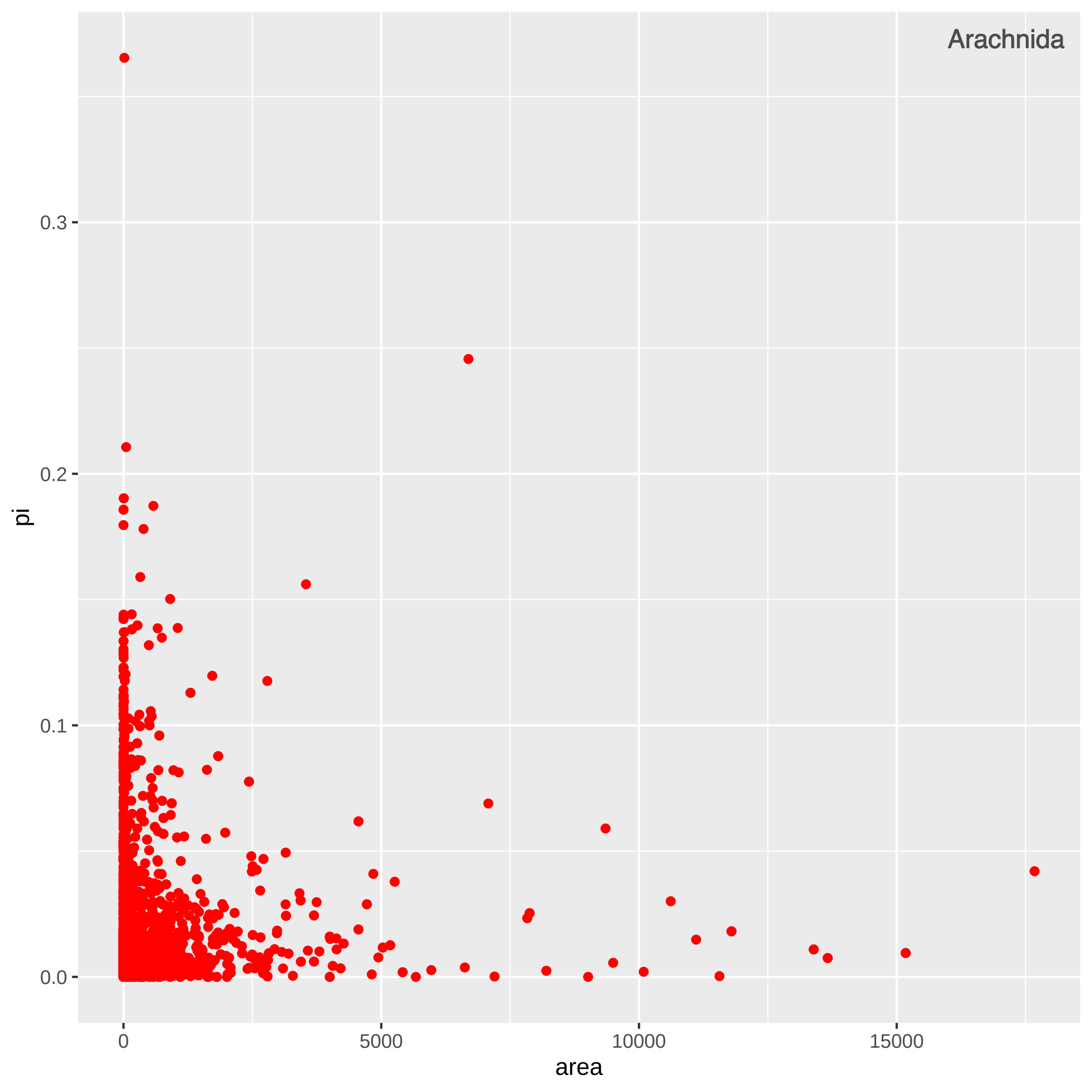

Arachnida
0.3
0.2
0.1
0.0
pi
0
5000
10000
15000
area

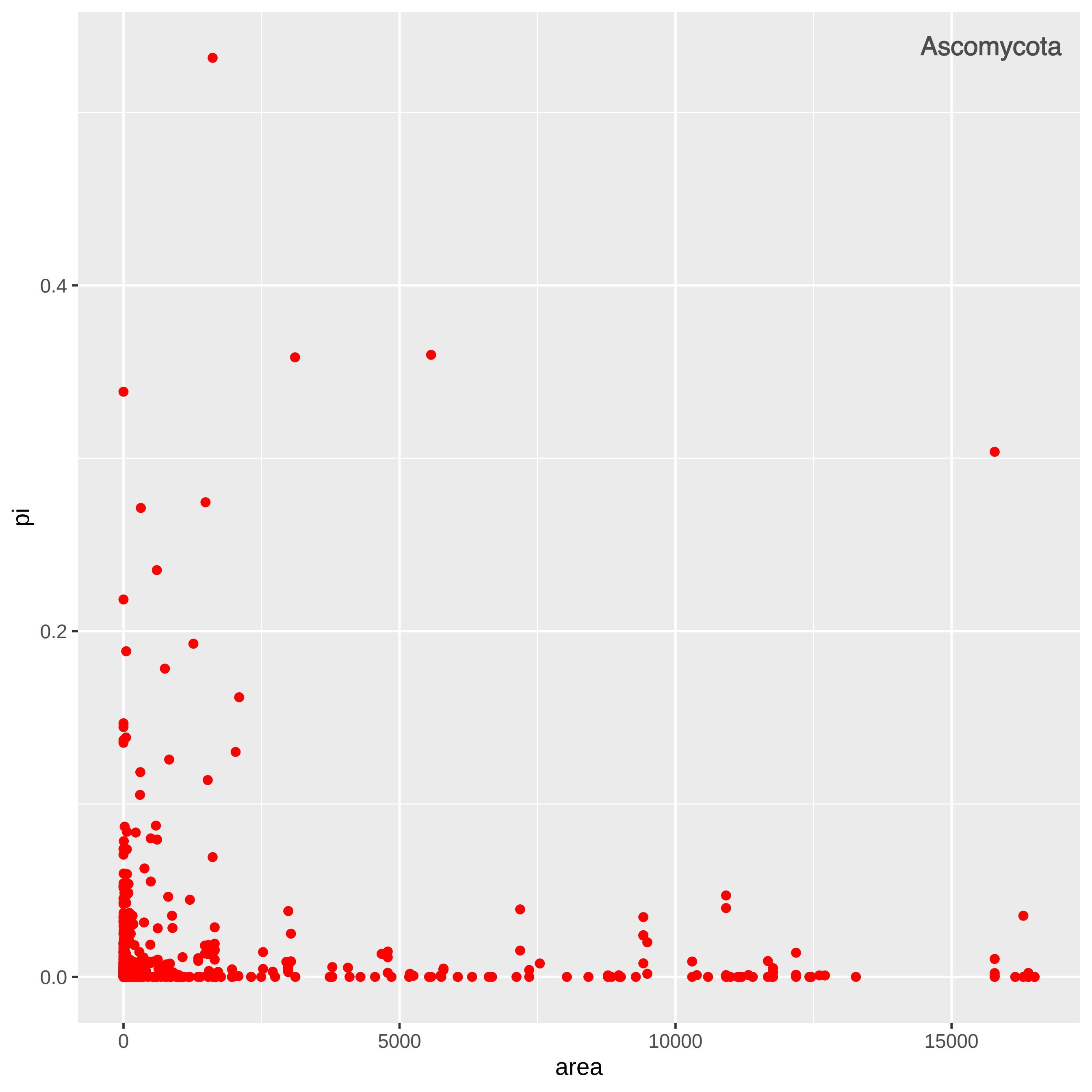

Ascomycota
pi
0.0
0.2
0.4
area
0
5000
10000
15000

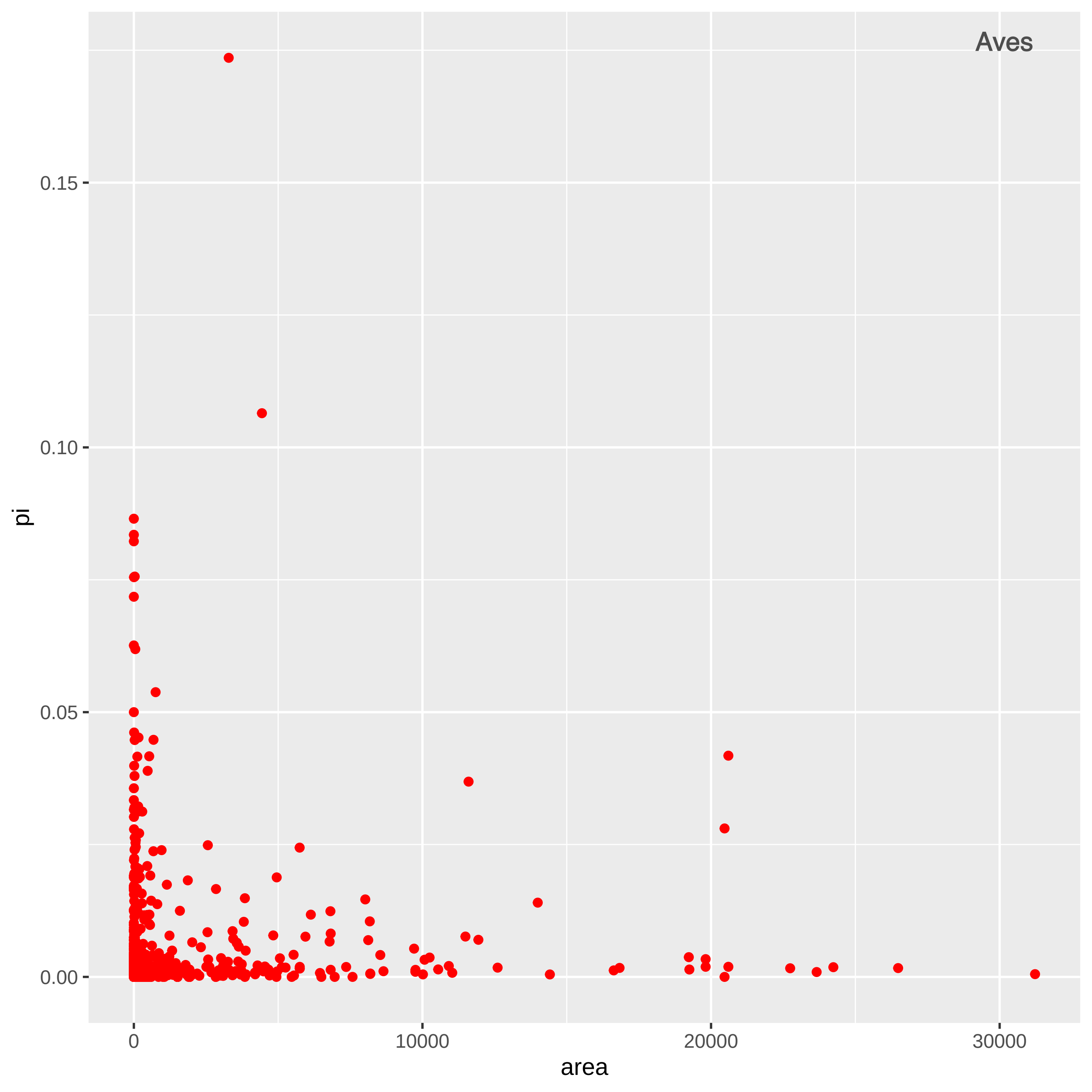

Aves
pi
0.15
0.10
0.05
0.00
0
10000
20000
30000
area

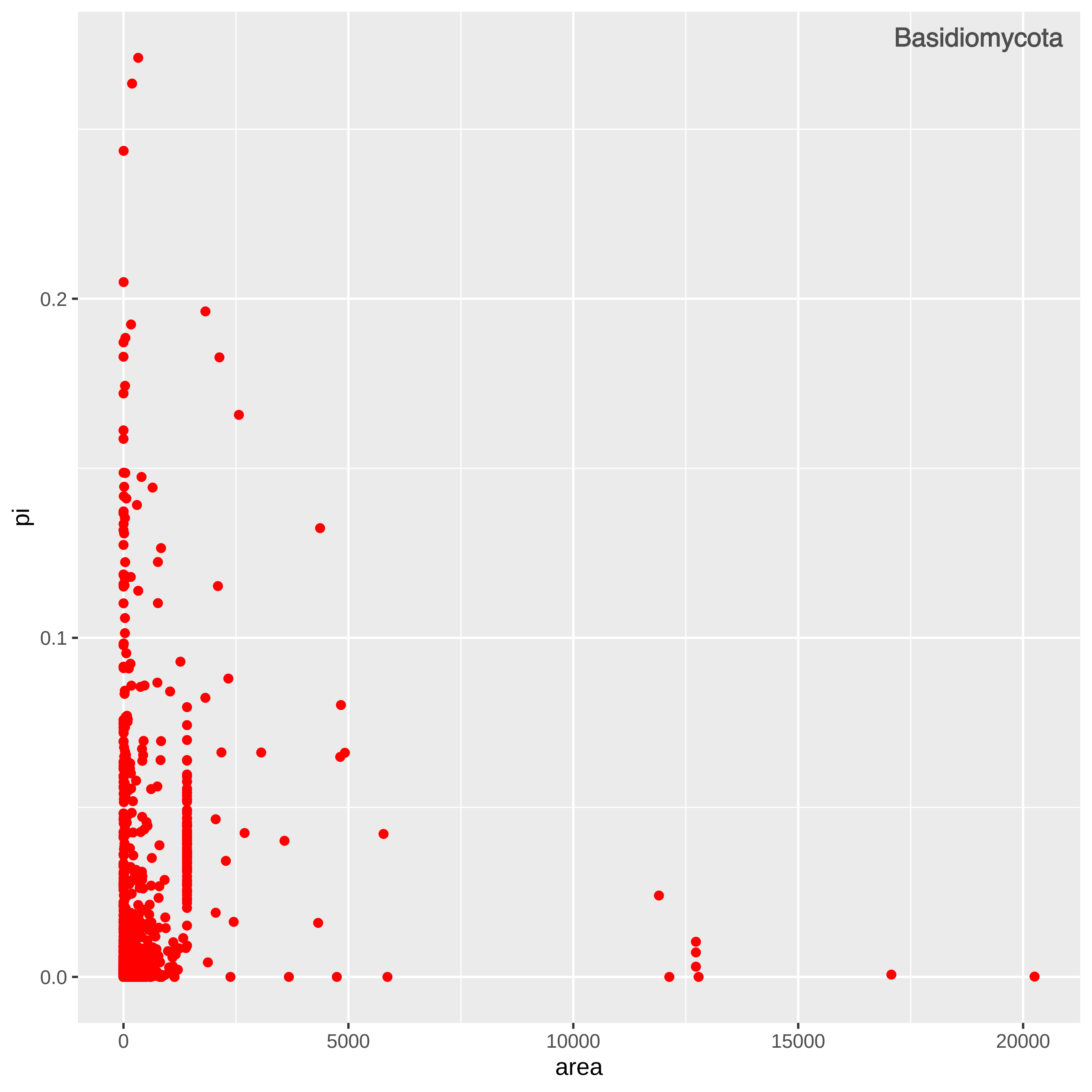
Basidiomycota
pi
0.0
0.1
0.2
area
0
5000
10000
15000
20000

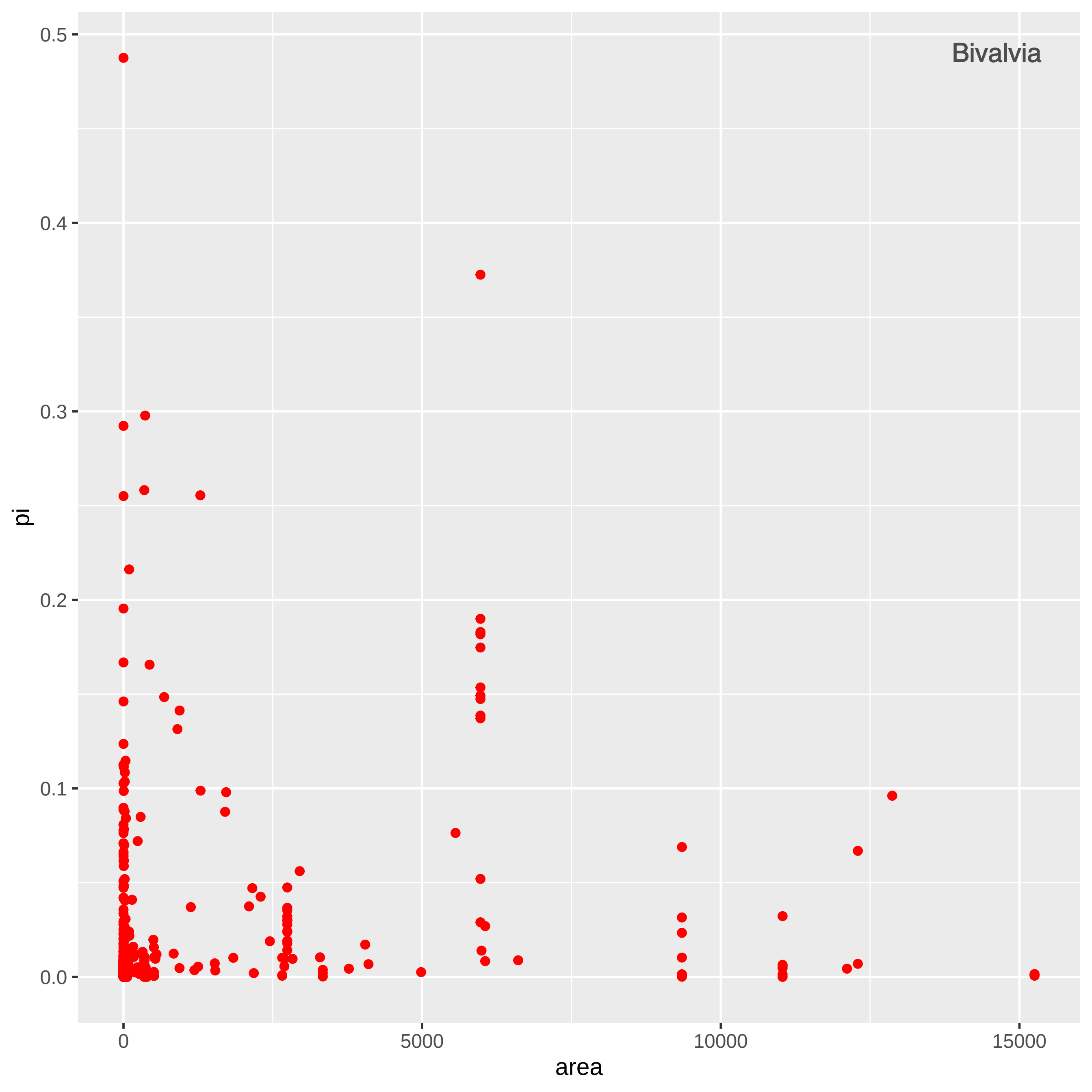

Bivalvia
pi
0.0
0.1
0.2
0.3
0.4
0.5
area
0
5000
10000
15000

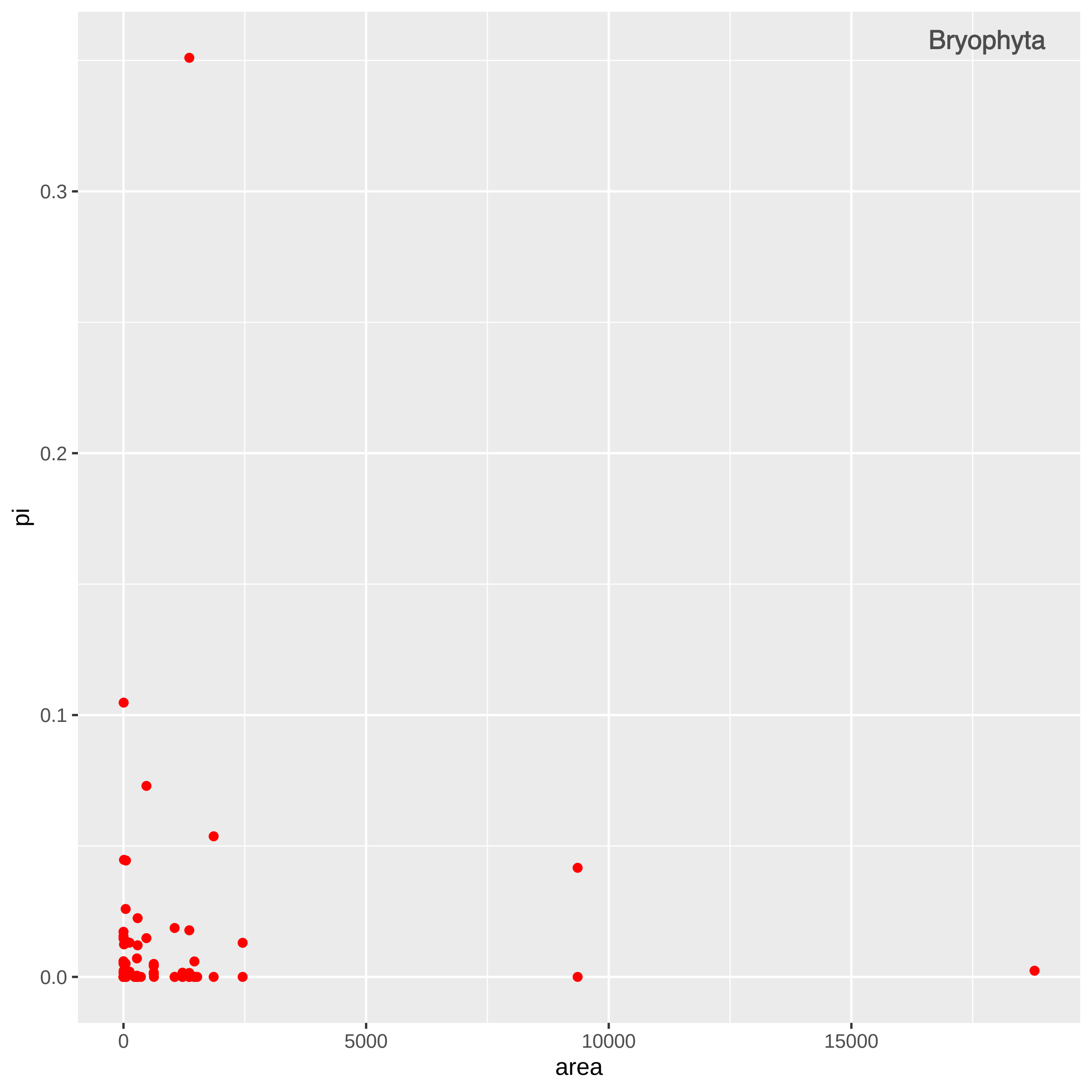
Bryophyta
pi
0.3
0.2
0.1
0.0
0
5000
10000
15000
area

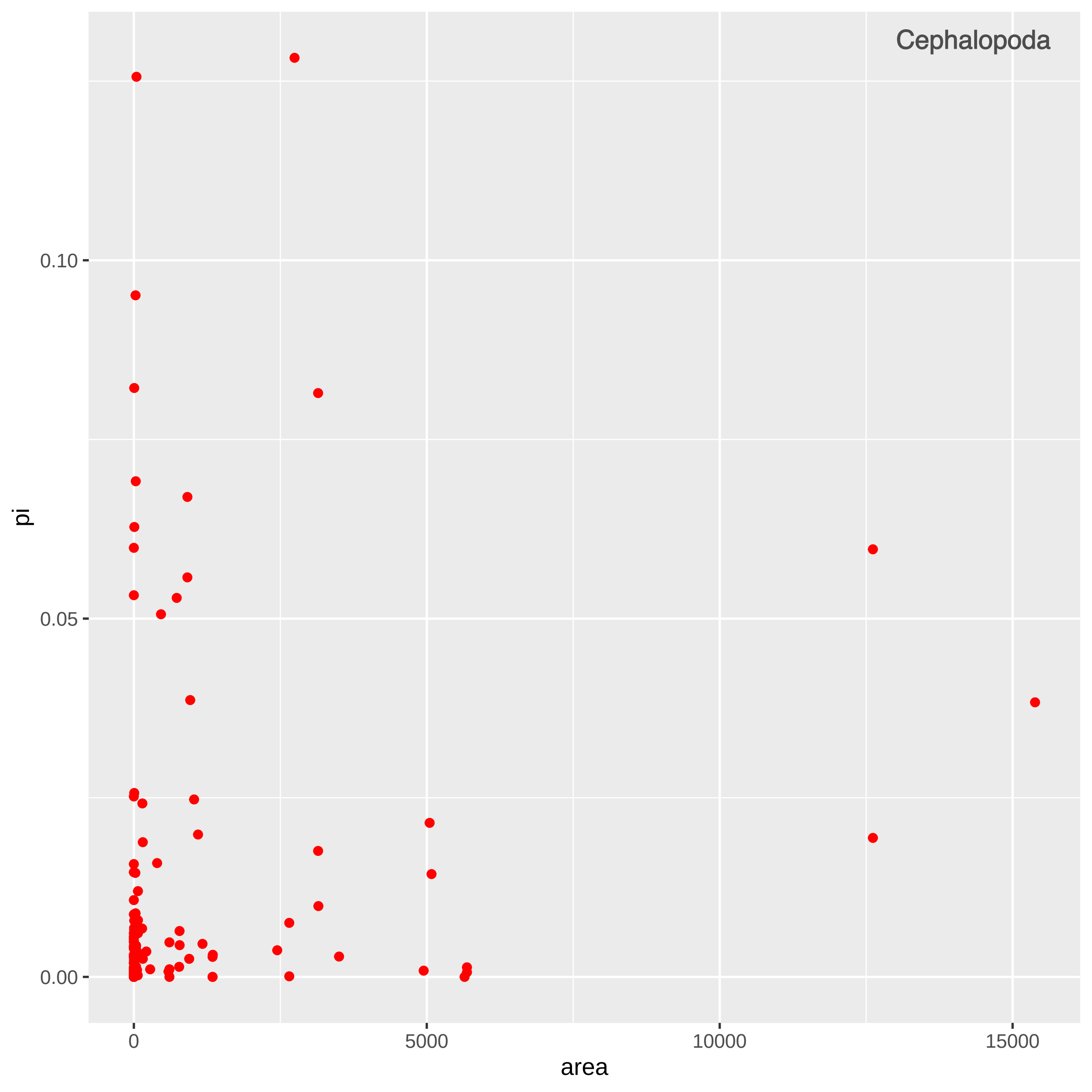
Cephalopoda
pi
0.10
0.05
0.00
0
5000
10000
15000
area

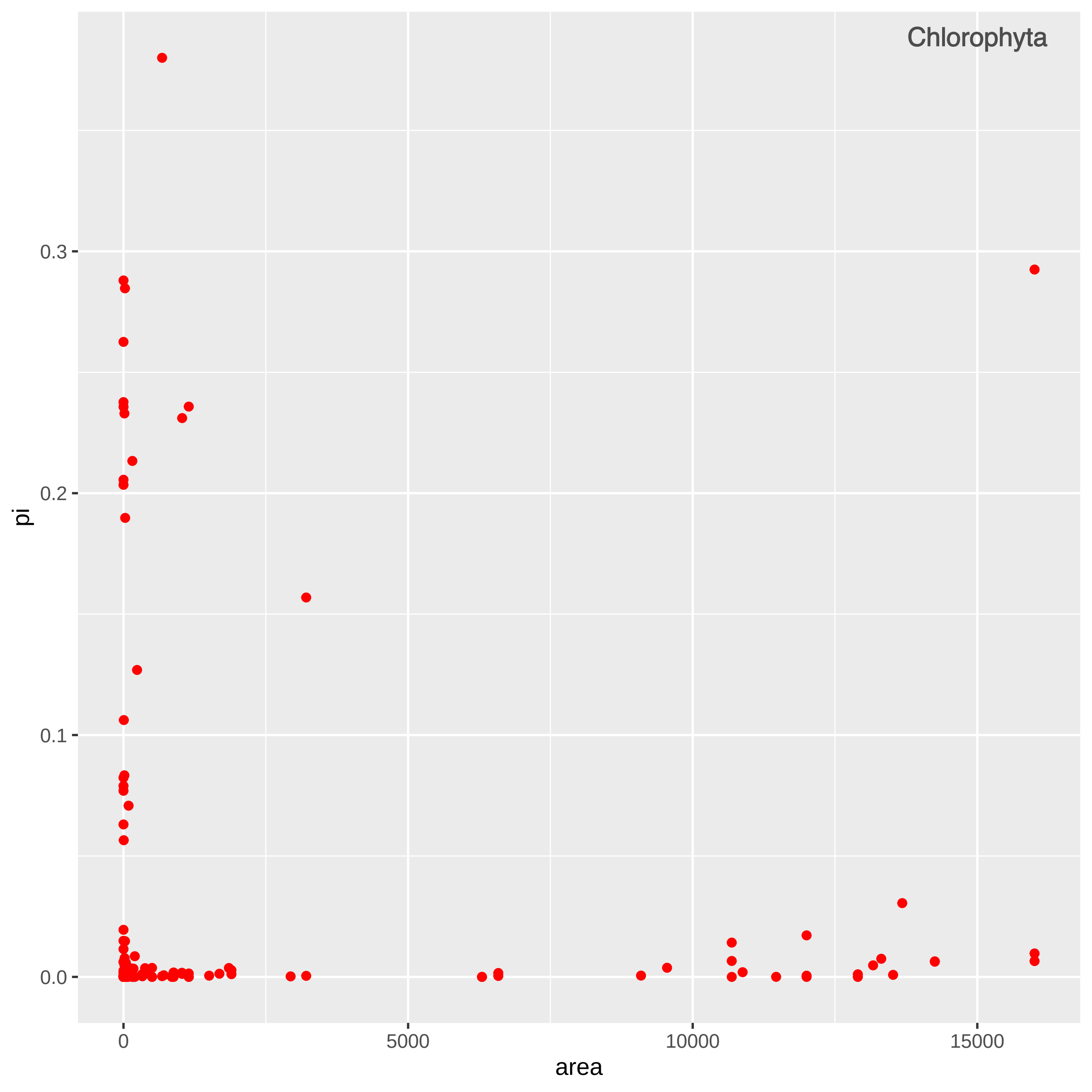

Chlorophyta
pi
0.0
0.1
0.2
0.3
area
0
5000
10000
15000

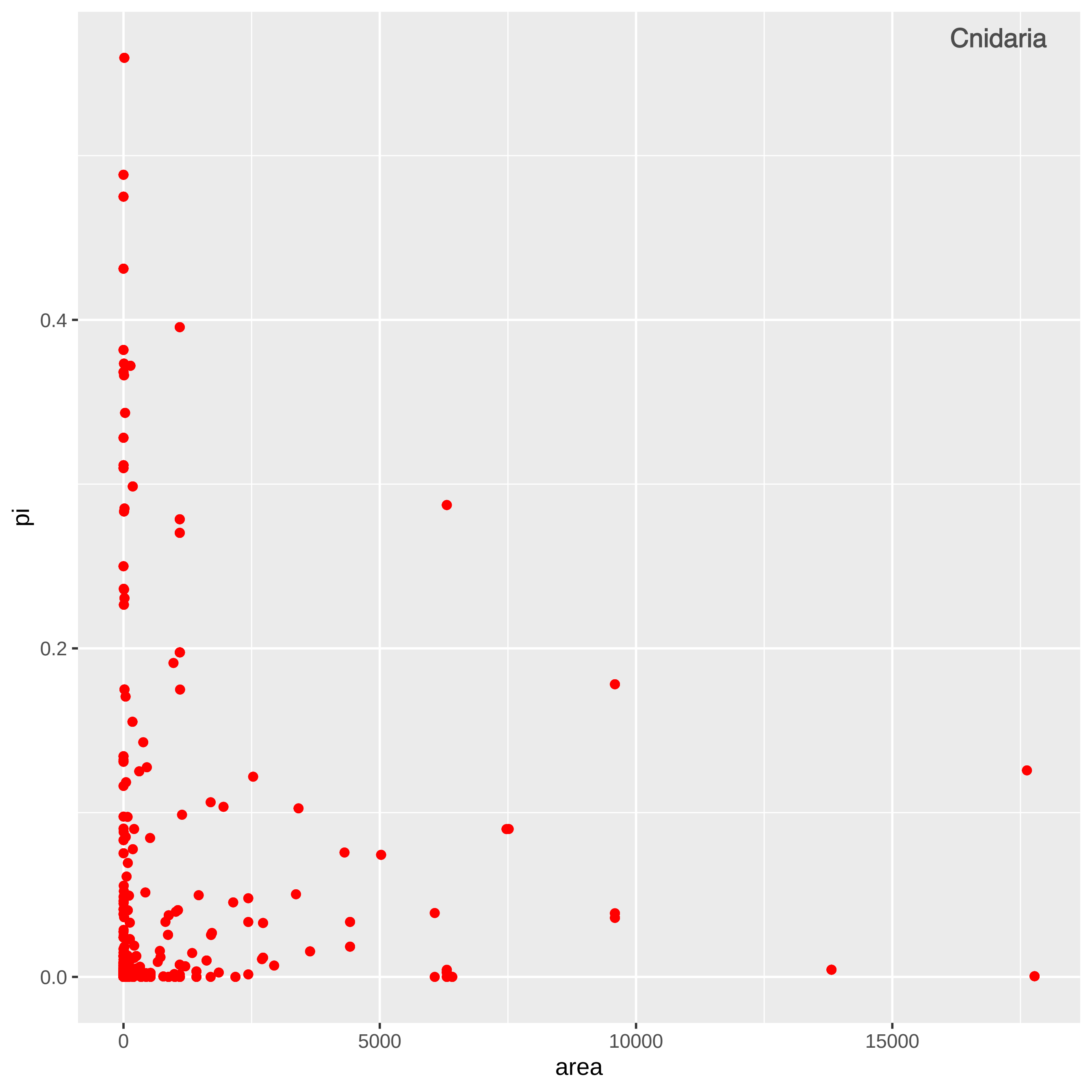
Cnidaria
pi
0.0
0.2
0.4
area
0
5000
10000
15000

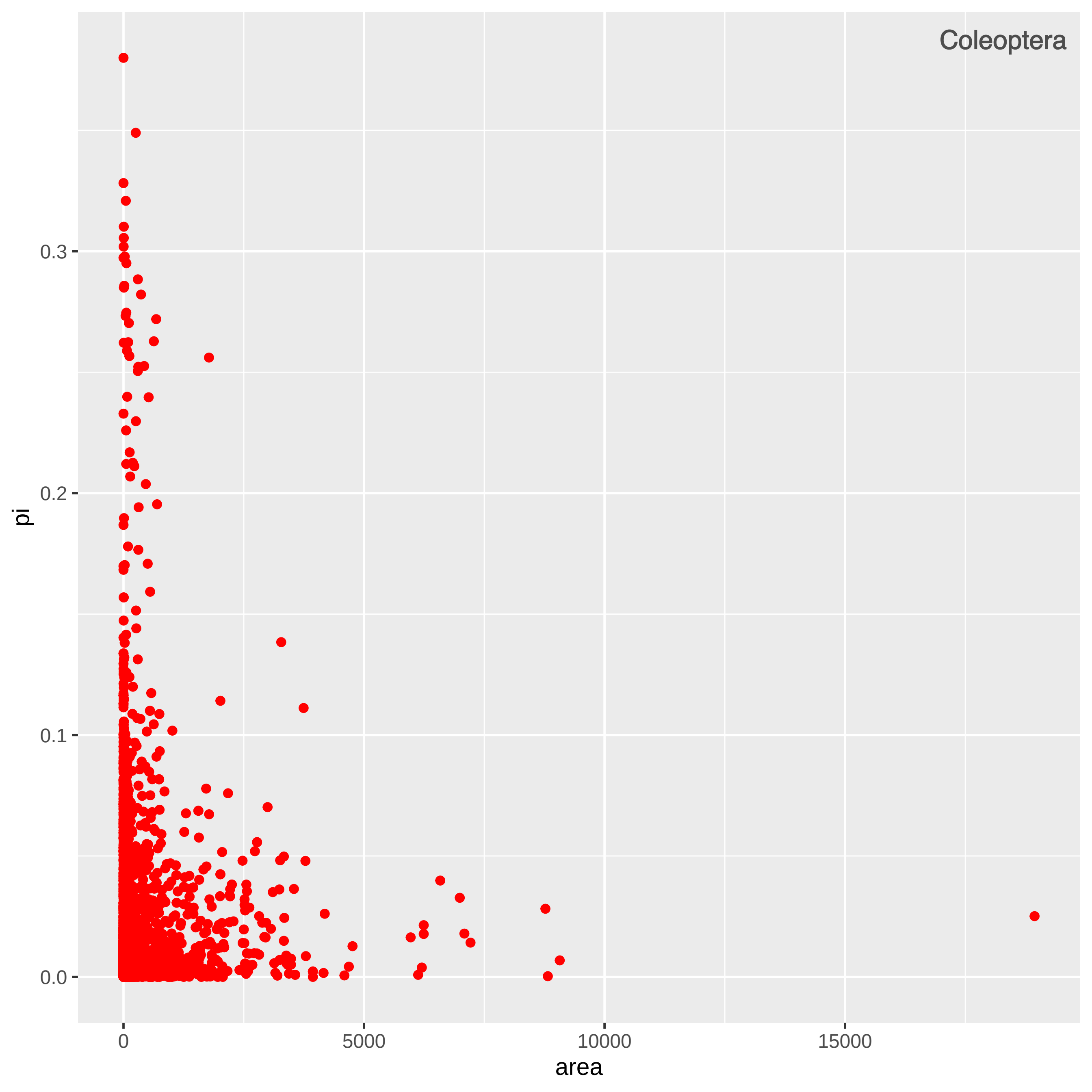

Coleoptera
pi
0.0
0.1
0.2
0.3
area
0
5000
10000
15000

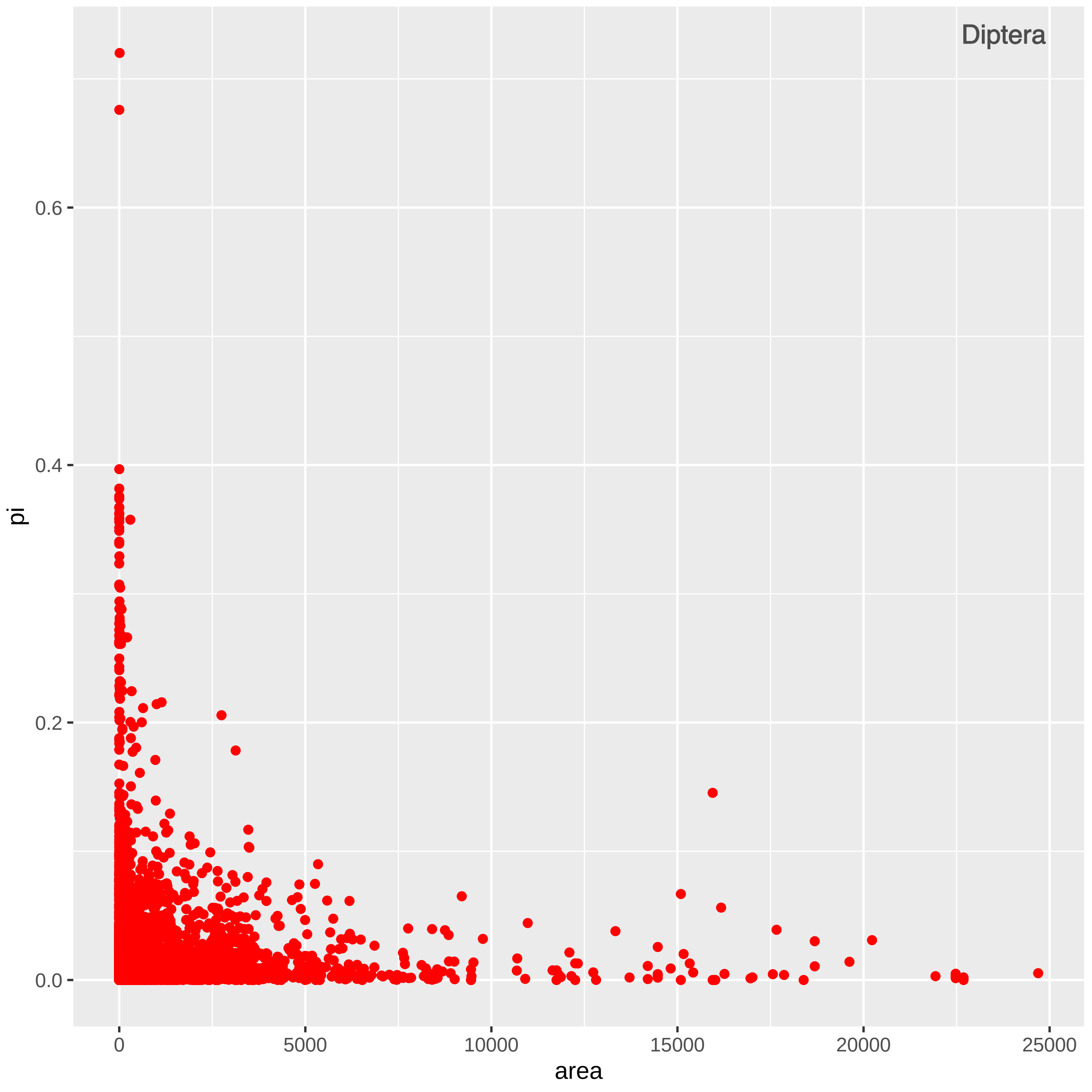

Diptera
pi
0.0
0.2
0.4
0.6
area
0
5000
10000
15000
20000
25000

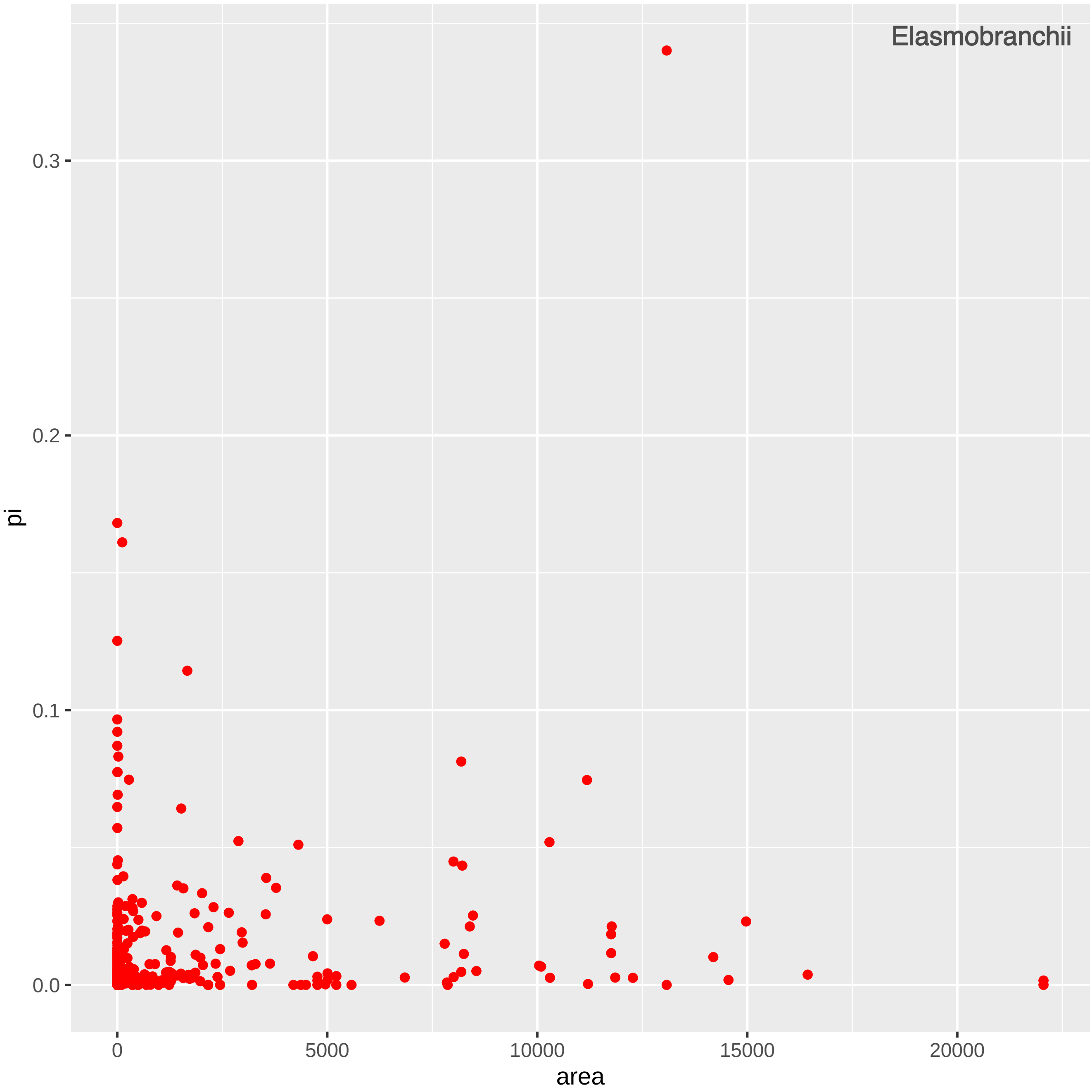
Elasmobranchii
pi
area
0.3
0.2
0.1
0.0
0
5000
10000
15000
20000

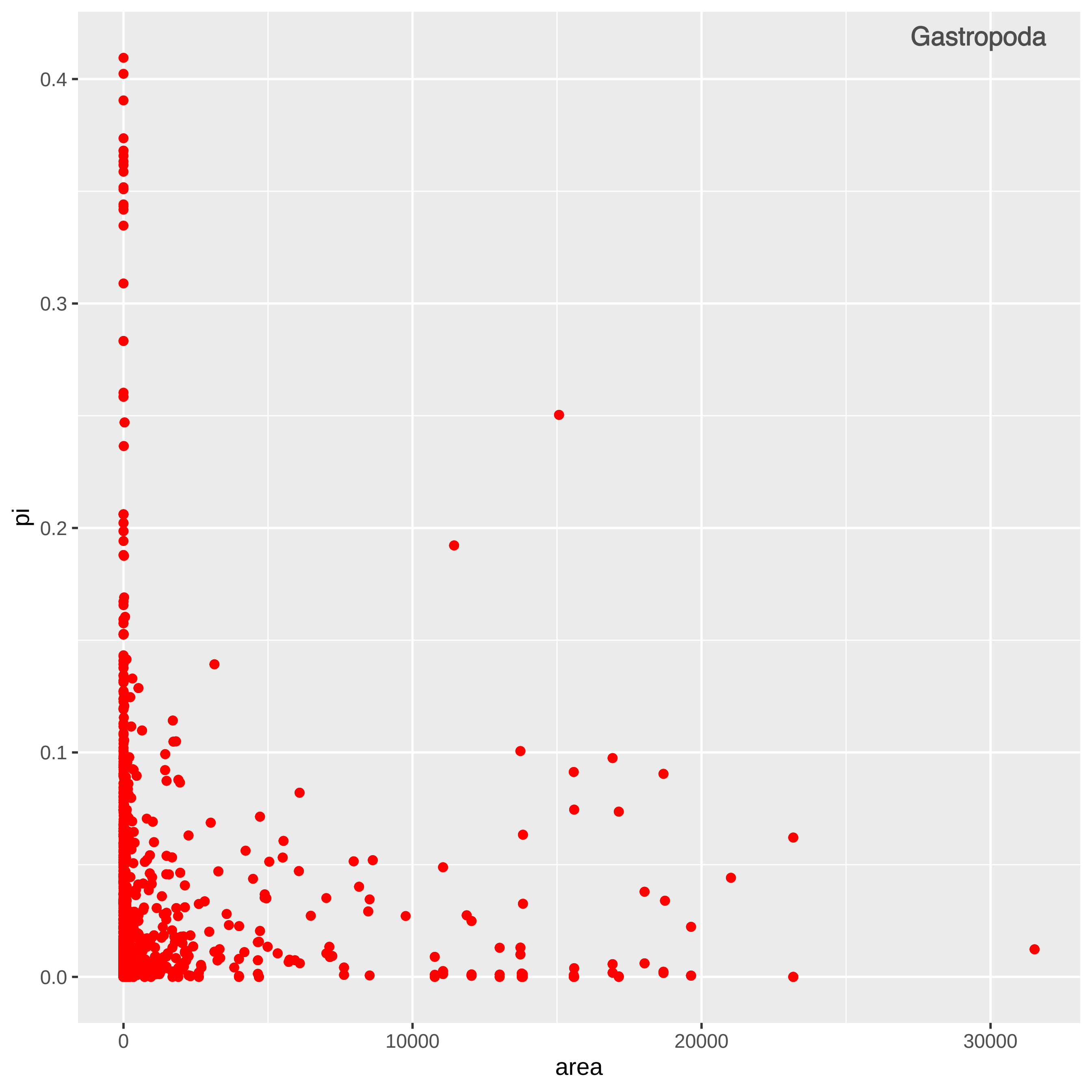
Gastropoda
0.4
0.3
0.2
0.1
0.0
pi
0
10000
20000
30000
area

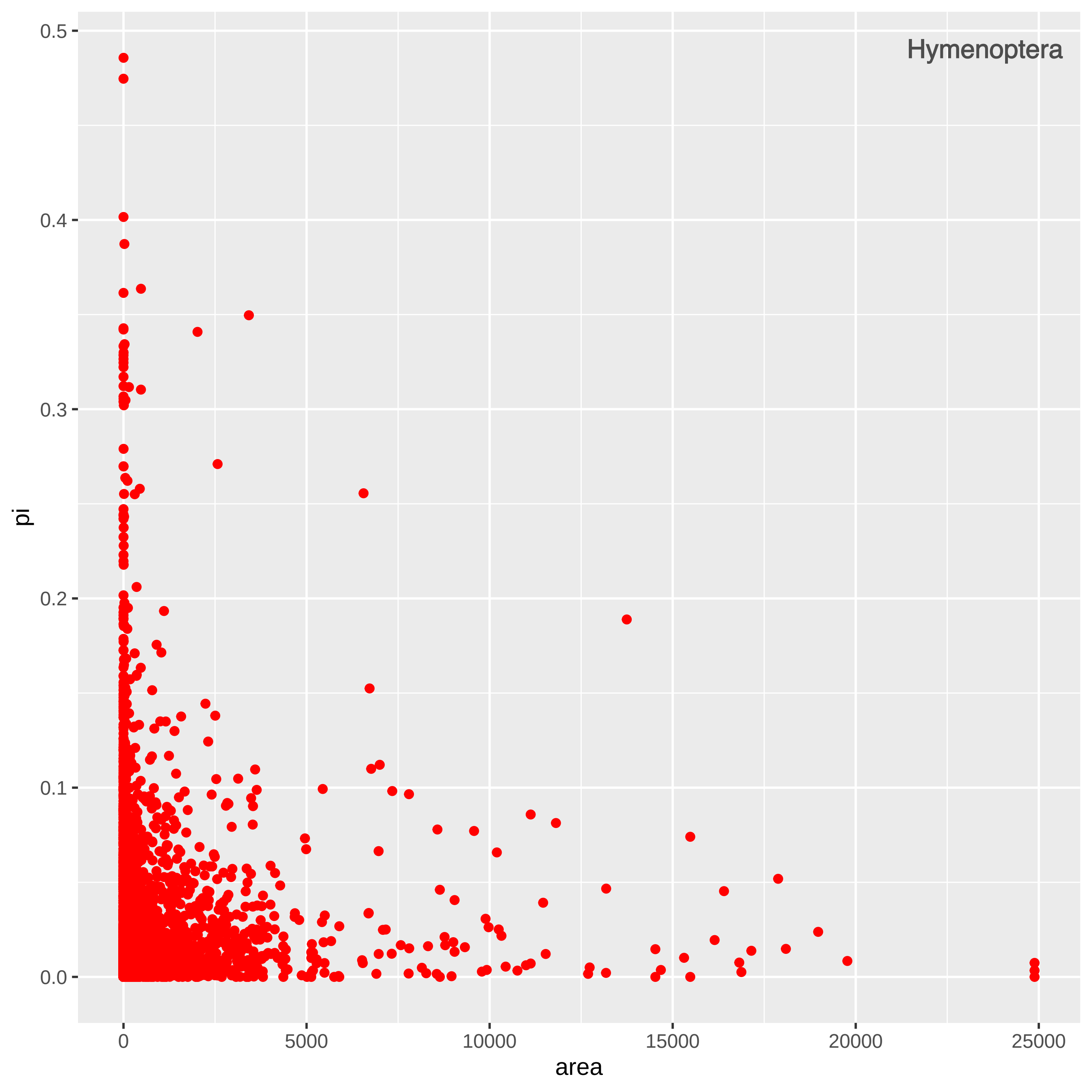
Hymenoptera
pi
0.5
0.4
0.3
0.2
0.1
0.0
0
5000
10000
15000
20000
25000
area

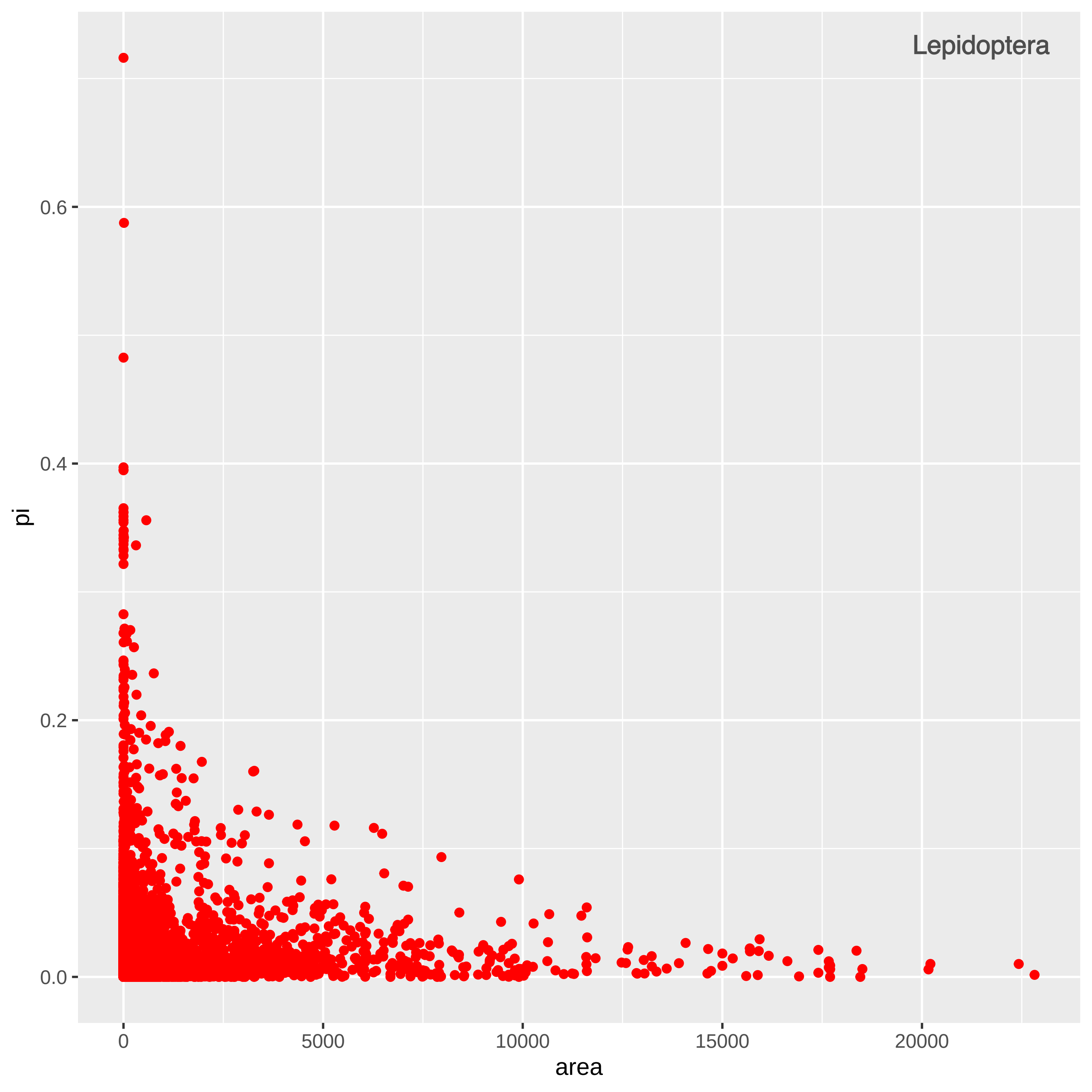
Lepidoptera
pi
0.6
0.4
0.2
0.0
0
5000
10000
15000
20000
area

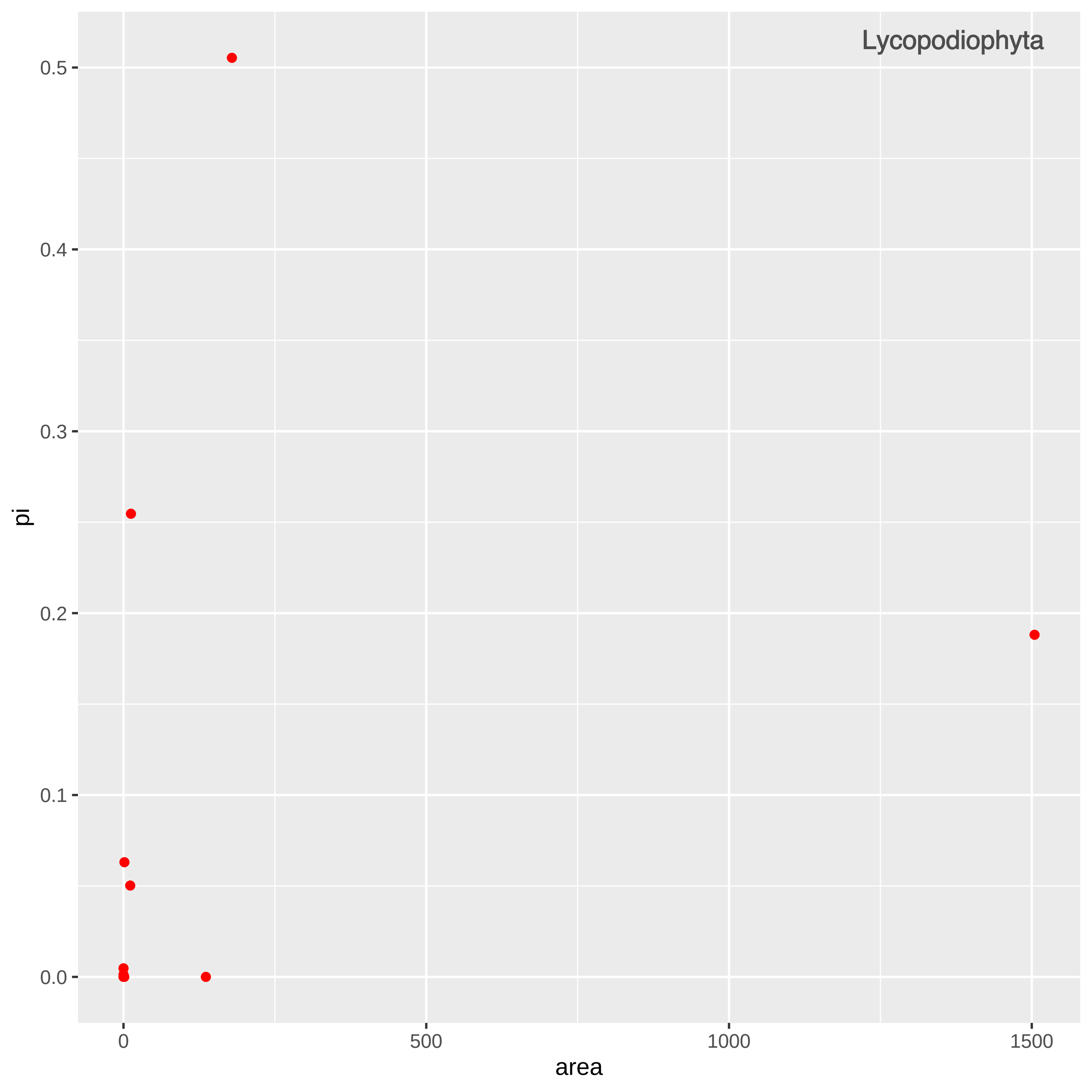
Lycopodiophyta
pi
area
0.0
0.1
0.2
0.3
0.4
0.5
0
500
1000
1500

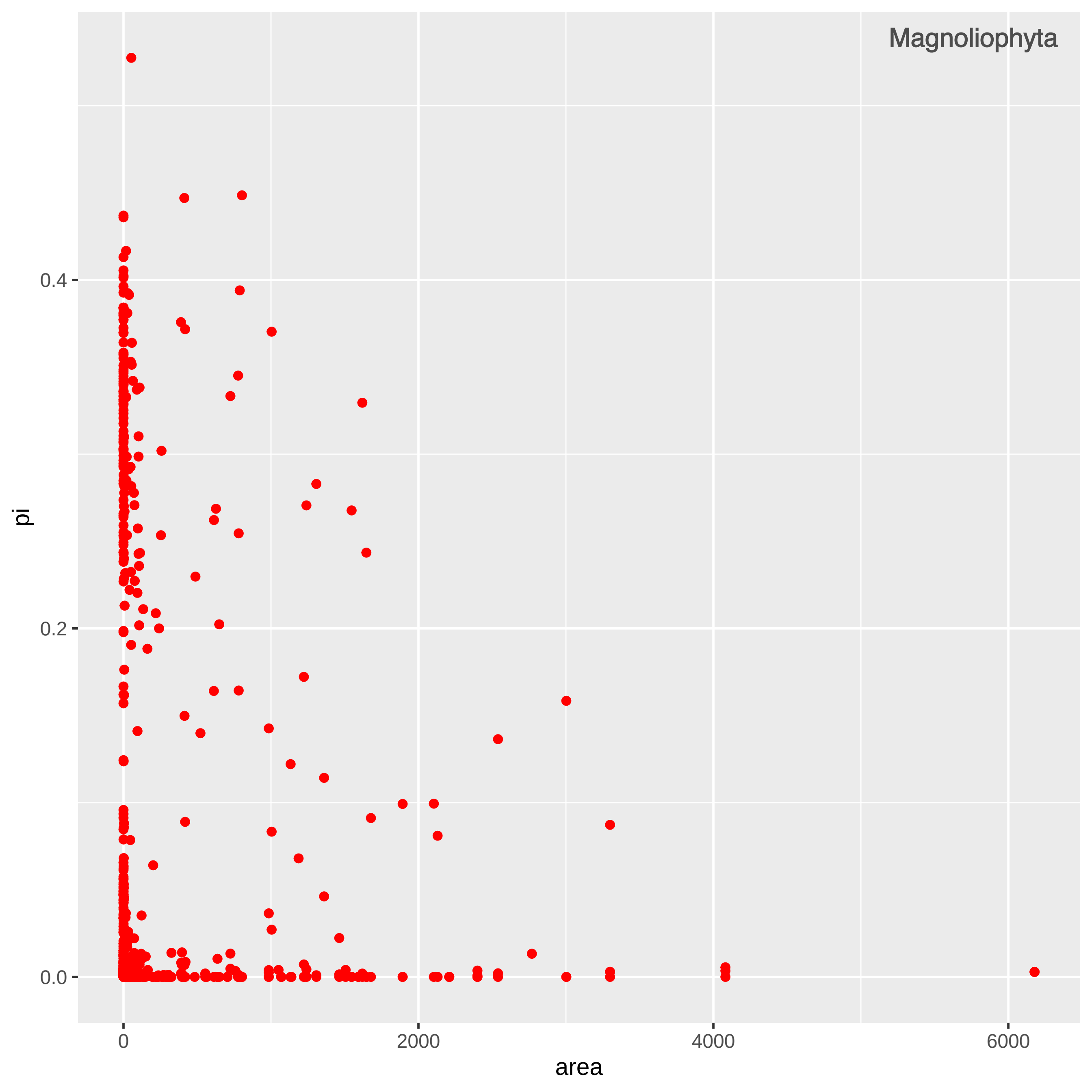
Magnoliophyta
pi
0.0
0.2
0.4
area
0
2000
4000
6000

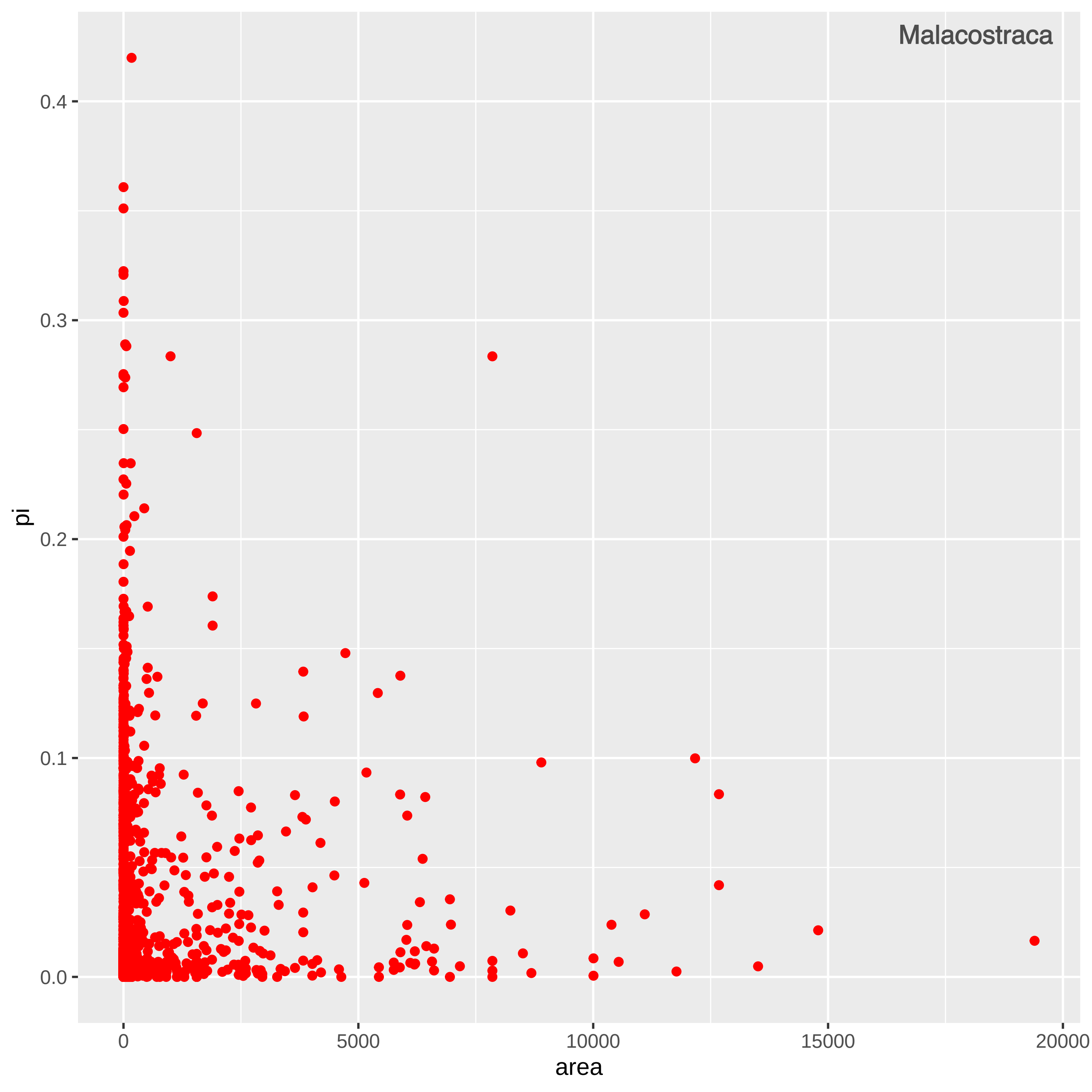
Malacostraca
pi
area
0.0
0.1
0.2
0.3
0.4
0
5000
10000
15000
20000

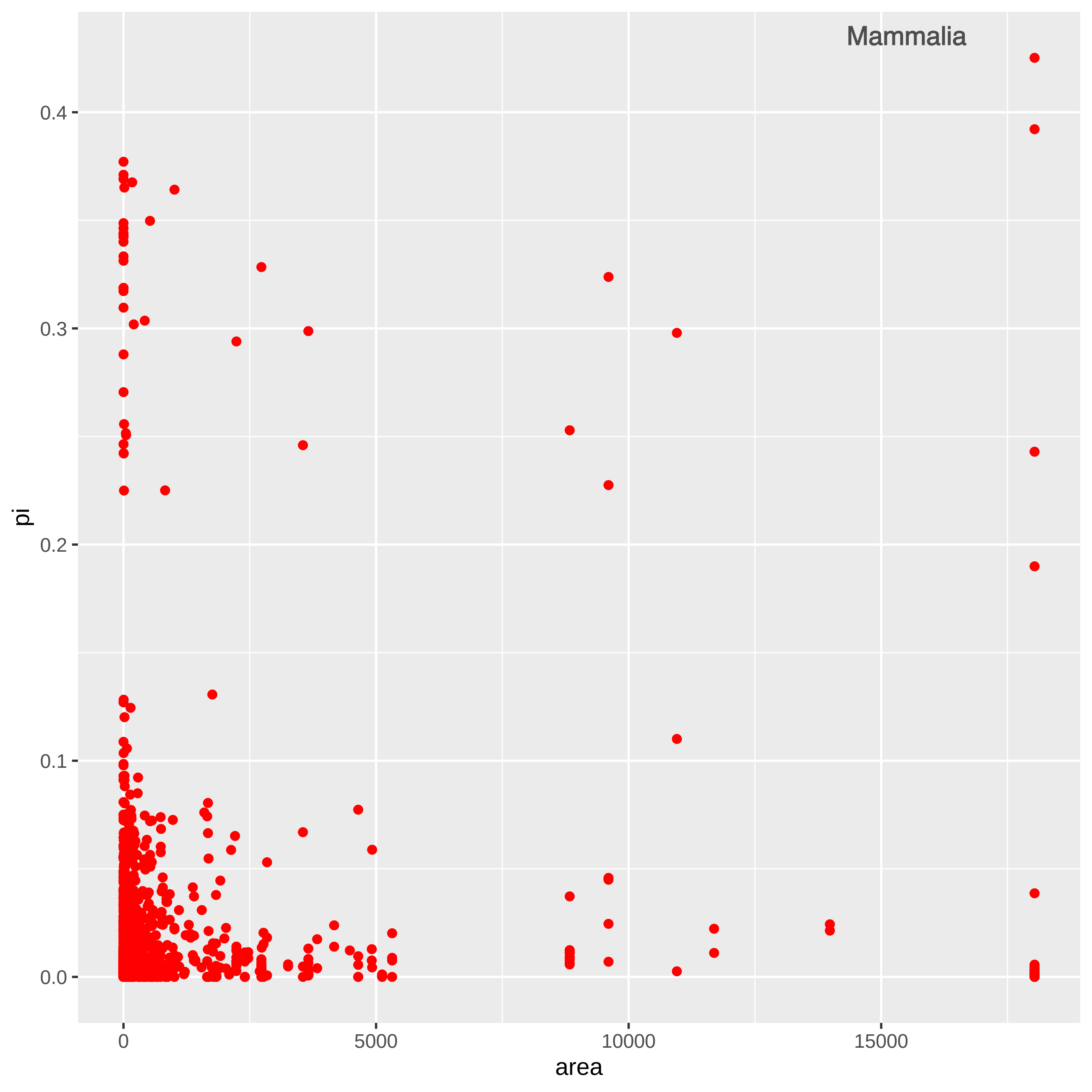
Mammalia
pi
0.0
0.1
0.2
0.3
0.4
area
0
5000
10000
15000

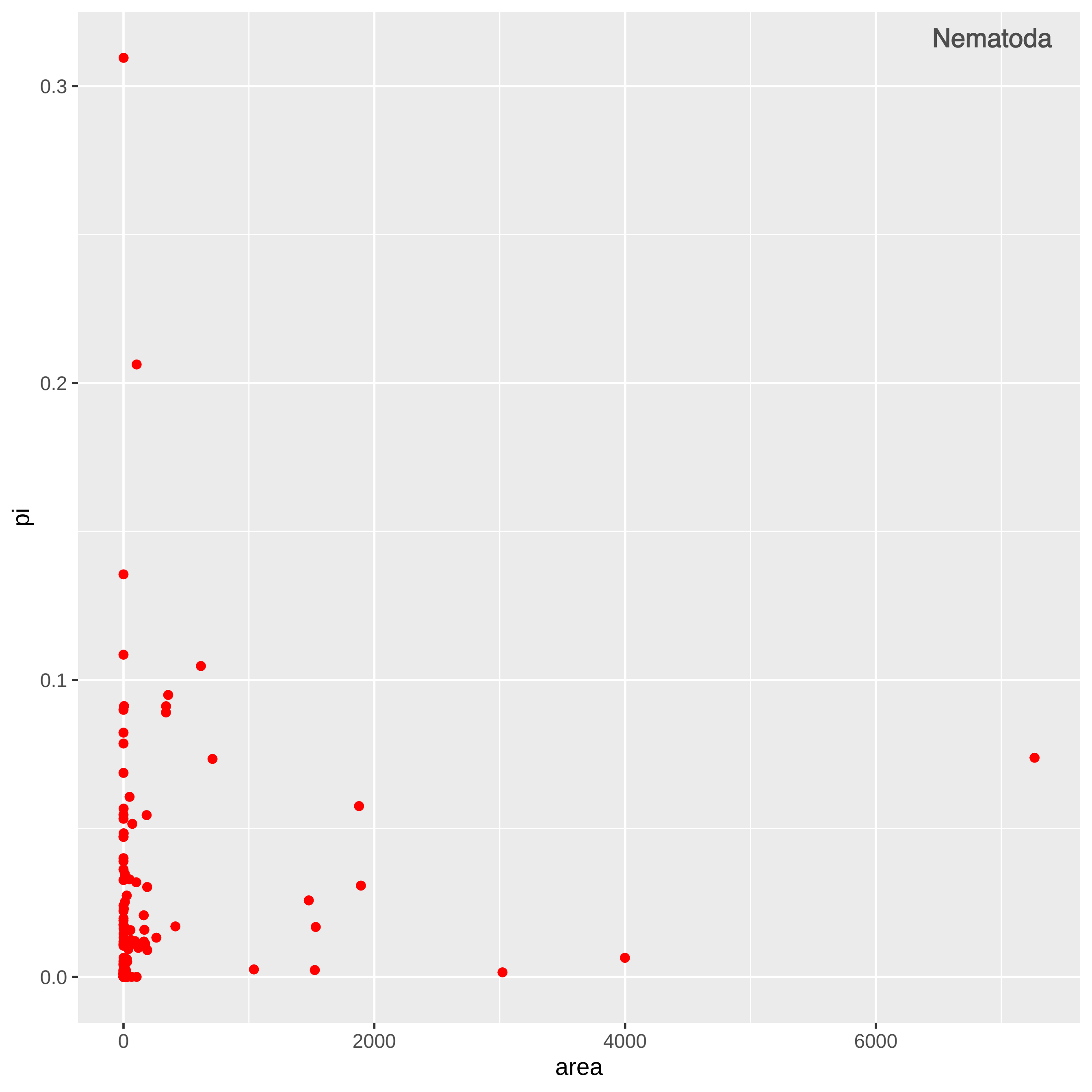

Nematoda
0.3
0.2
0.1
0.0
pi
0
2000
4000
6000
area

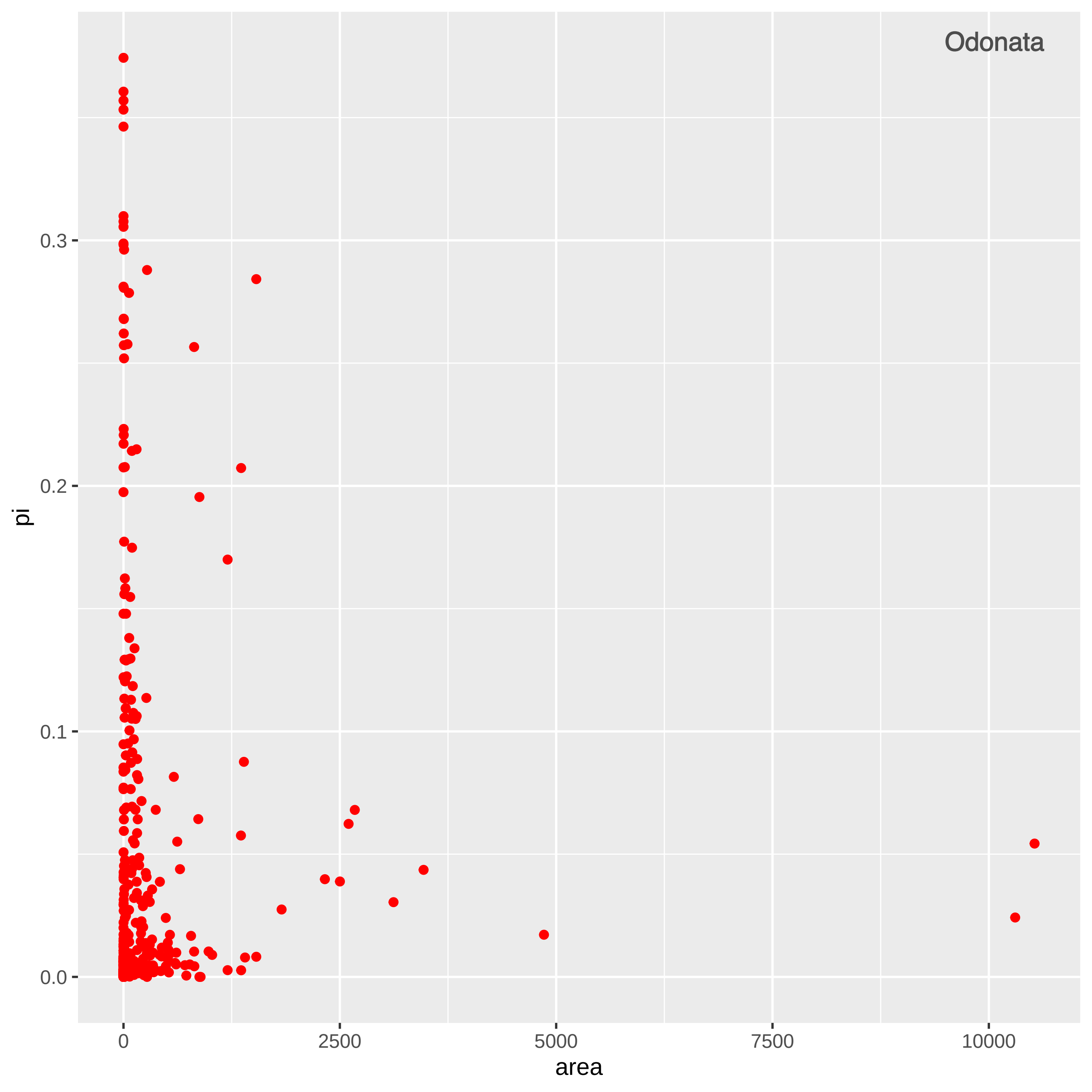
Odonata
0.3
0.2
0.1
0.0
pi
0
2500
5000
7500
10000
area

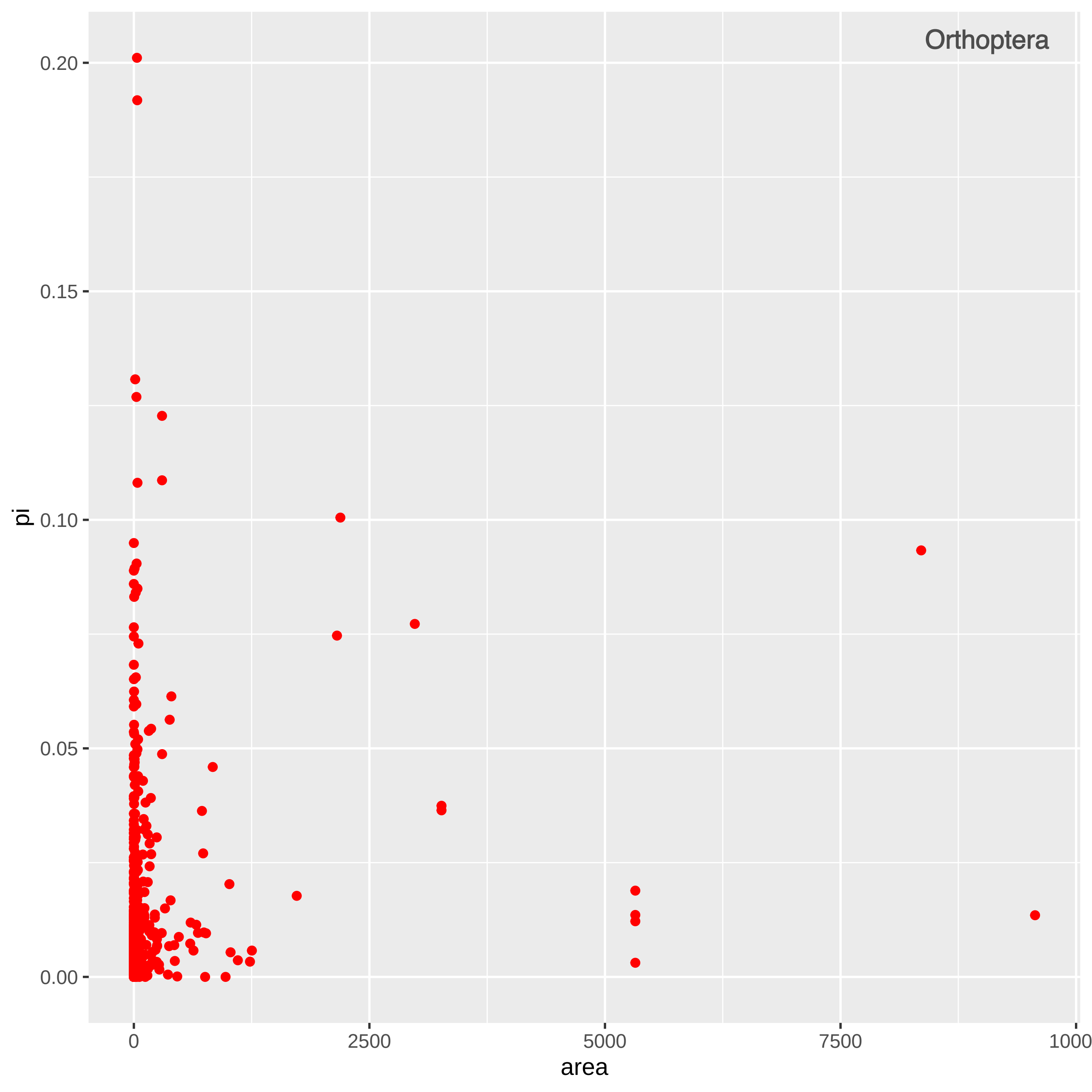

Orthoptera
pi
0.20
0.15
0.10
0.05
0.00
0
2500
5000
7500
1000
area

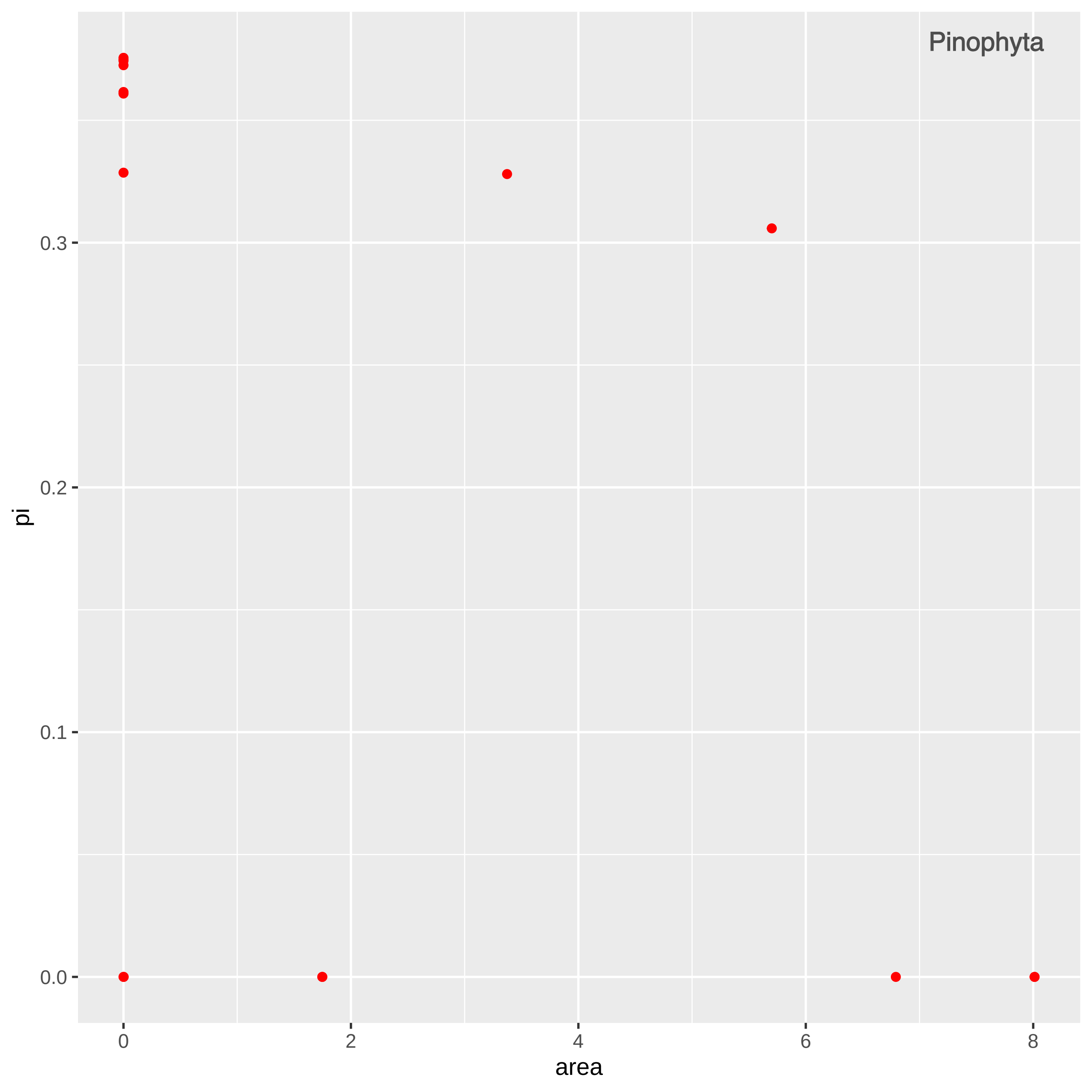
Pinophyta
pi
0.0
0.1
0.2
0.3
area
0
2
4
6
8

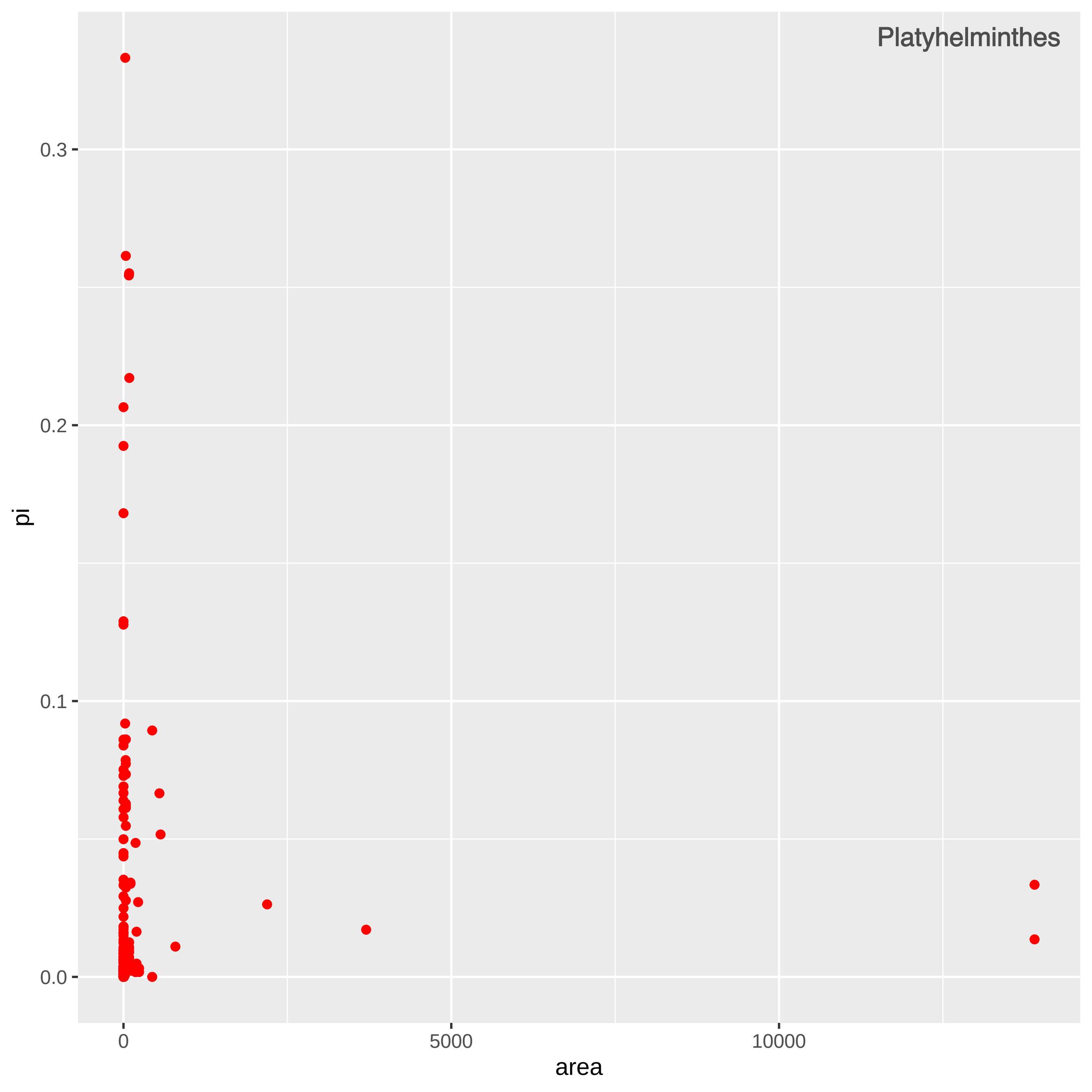

Platyhelminthes
0.3
0.2
0.1
0.0
pi
0
5000
10000
area

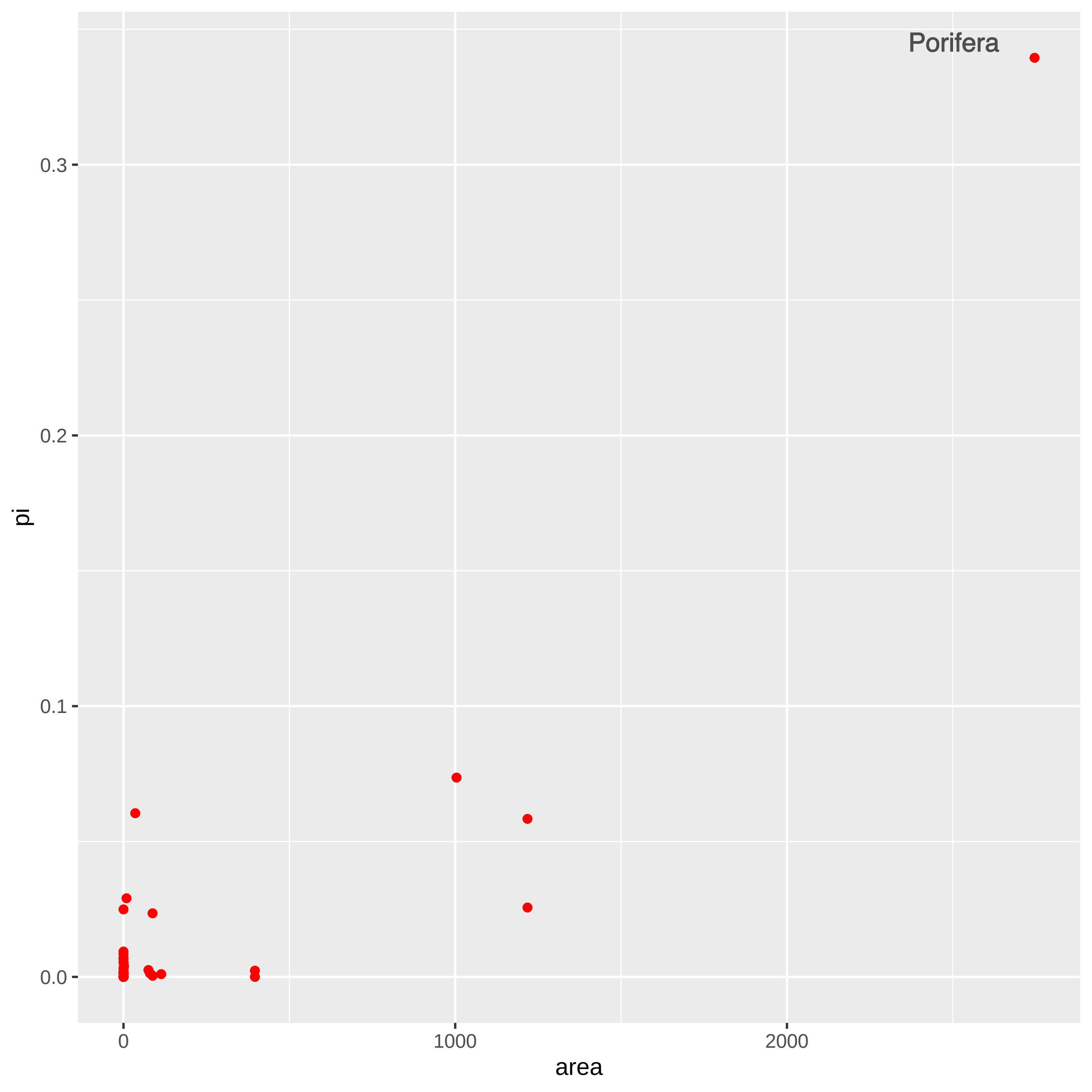

Porifera
0.3
0.2
0.1
0.0
pi
0
1000
2000
area

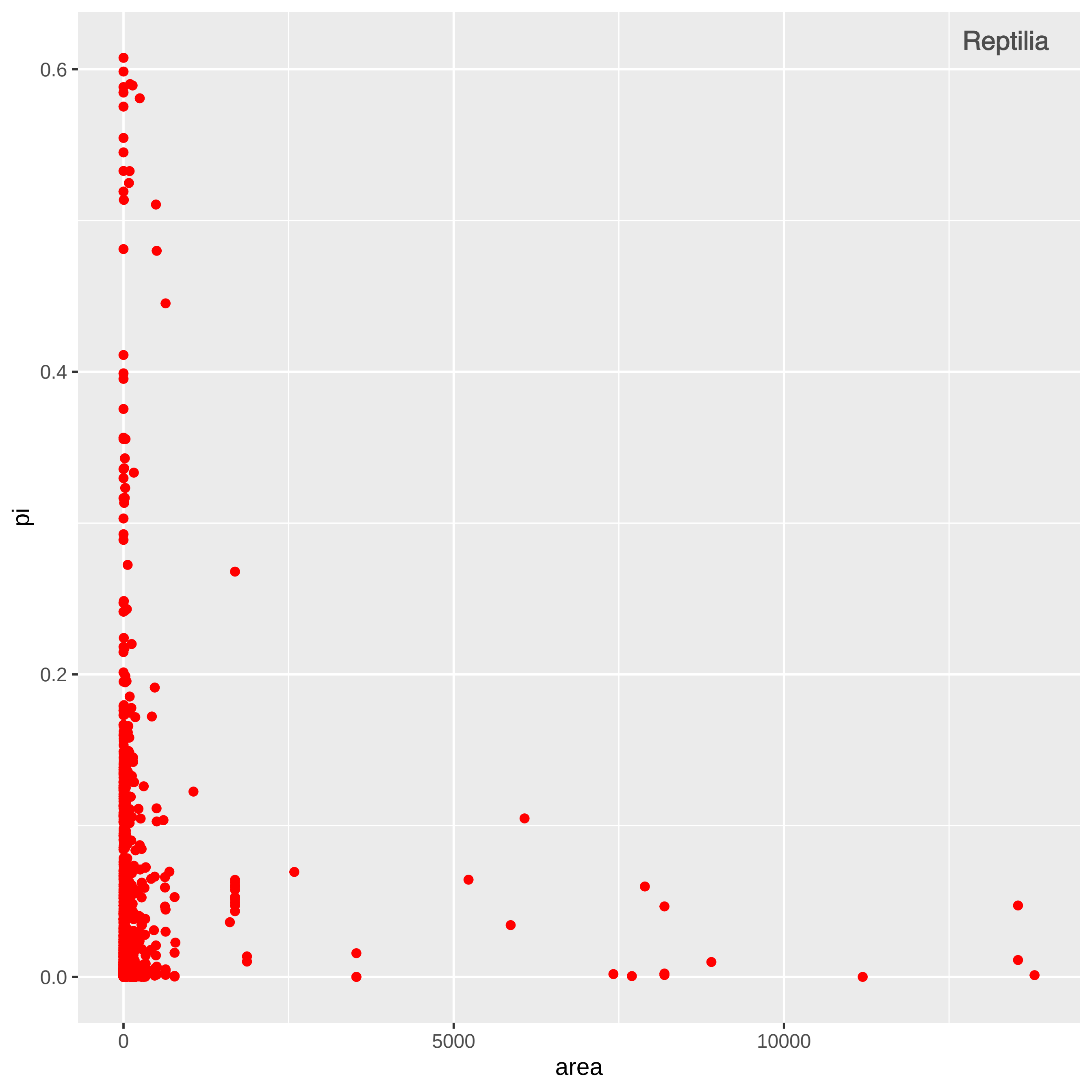
Reptilia
pi
0.6
0.4
0.2
0.0
area
0
5000
10000

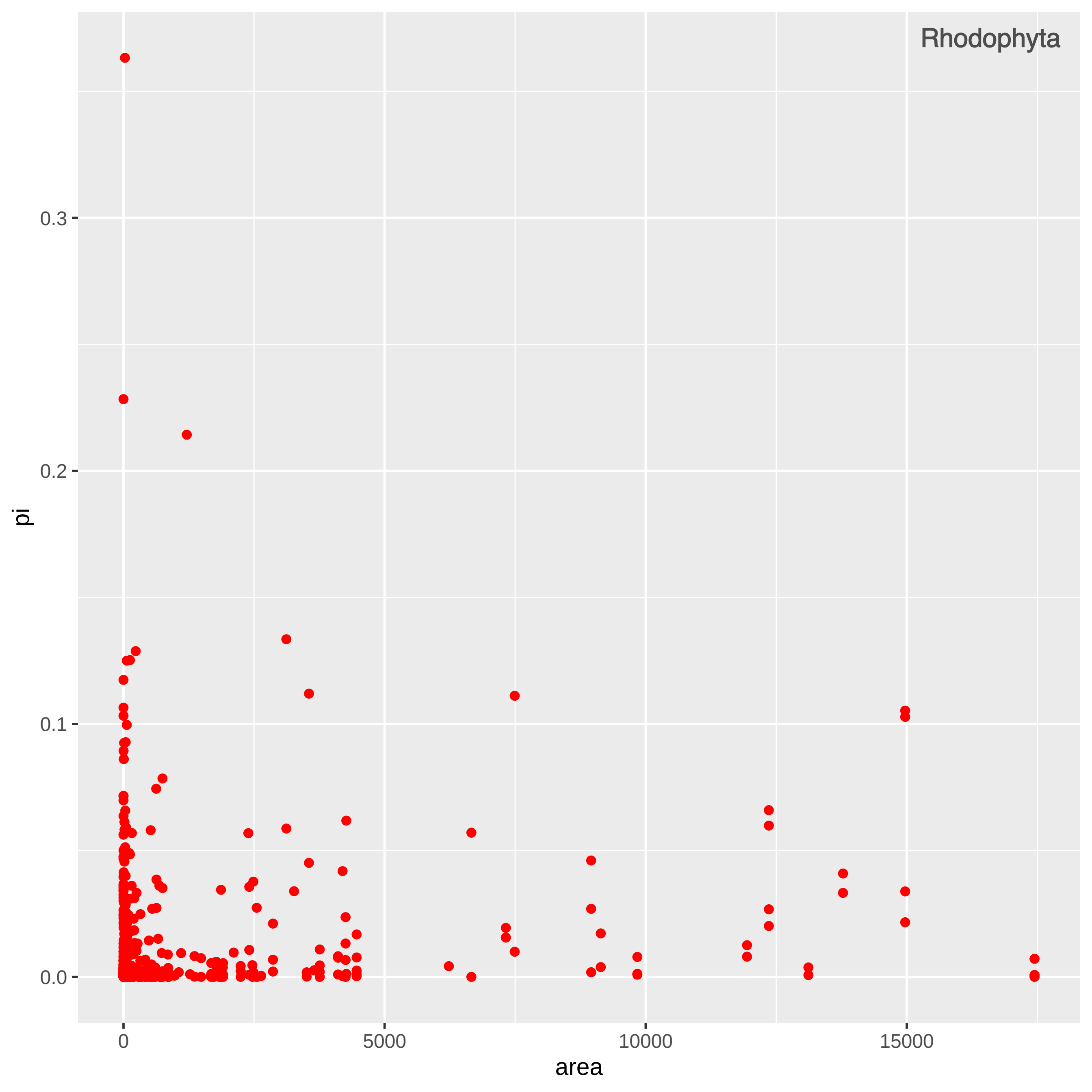

Rhodophyta
pi
0.0
0.1
0.2
0.3
area
0
5000
10000
15000

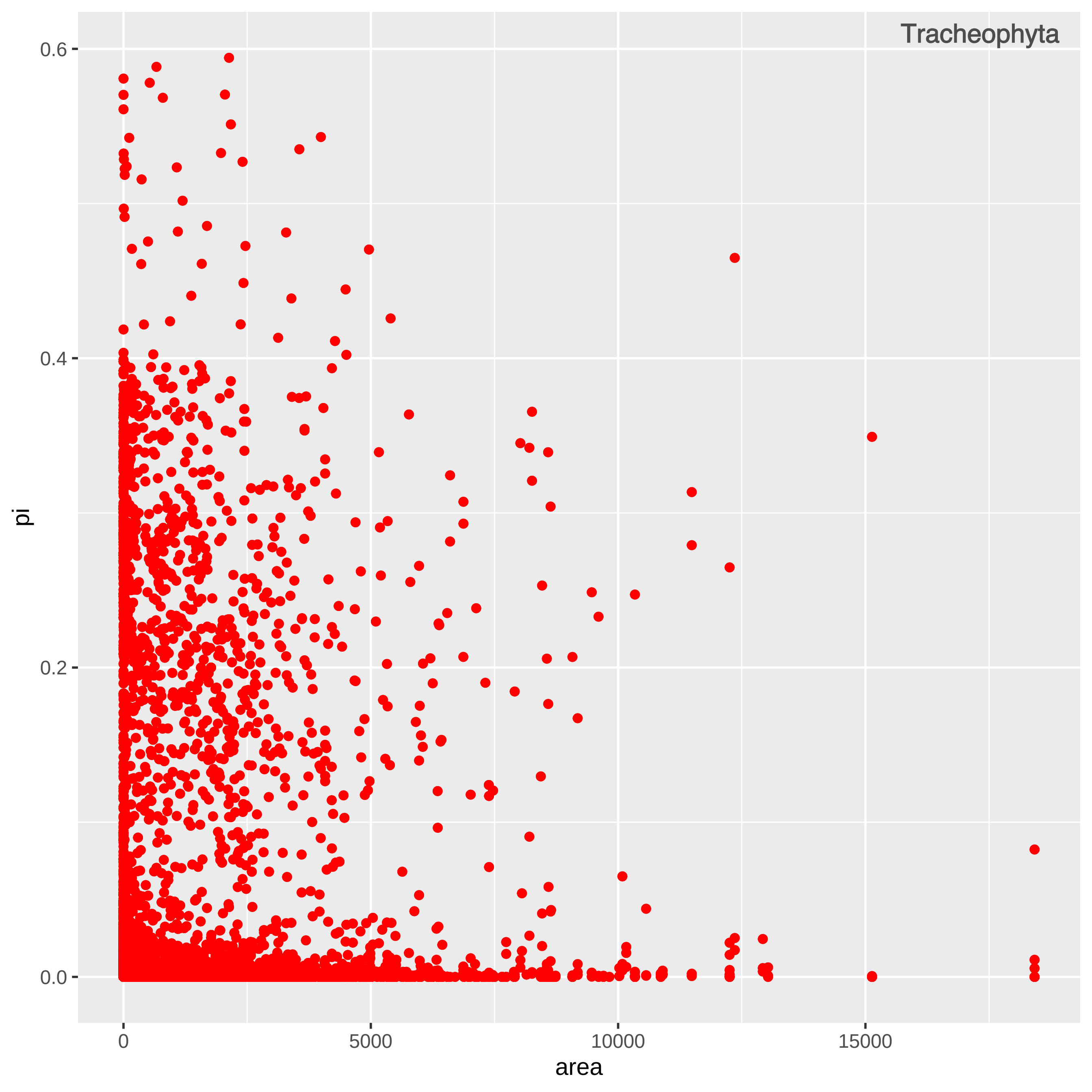

Tracheophyta
pi
area
0.6
0.4
0.2
0.0
0
5000
10000
15000
